# Personalized Dietary Recommendations Based on Lipid-Related Genetic Variants: A Systematic Review

**DOI:** 10.3389/fnut.2022.830283

**Published:** 2022-03-21

**Authors:** Yolanda E. Pérez-Beltrán, Ingrid Rivera-Iñiguez, Karina Gonzalez-Becerra, Naomi Pérez-Naitoh, Juscelino Tovar, Sonia G. Sáyago-Ayerdi, Edgar J. Mendivil

**Affiliations:** ^1^Laboratorio Integral de Investigación en Alimentos, Instituto Tecnológico de Tepic/Instituto Nacional de México, Tepic, Mexico; ^2^Departamento de Reproducción Humana, Crecimiento y Desarrollo Infantil, Centro Universitario de Ciencias de la Salud, Universidad de Guadalajara, Guadalajara, Mexico; ^3^Departamento de Ciencias Médicas y de la Vida, Centro Universitario de la Ciénega, Instituto de Investigación en Genética Molecular, Universidad de Guadalajara, Guadalajara, Mexico; ^4^Grupo de Investigación en Nutrición y Ciencias de los Alimentos, Departamento de Psicología, Educación y Salud, ITESO, Universidad Jesuita de Guadalajara, Tlaquepaque, Mexico; ^5^Departamento de Salud, Universidad Iberoamericana (IBERO), Mexico City, Mexico; ^6^Department of Food Technology, Engineering, and Nutrition, Lund University, Lund, Sweden

**Keywords:** nutritional genetics, nutrigenomics, dyslipidemia, obesity, polymorphism, gene-diet interaction

## Abstract

**Background:**

Obesity and dyslipidemias are risk factors for developing cardiovascular diseases, the leading causes of morbidity and mortality worldwide. The pathogenesis of these diseases involves environmental factors, such as nutrition, but other aspects like genetic polymorphisms confer susceptibility to developing obesity and dyslipidemias. In this sense, nutrigenetics is being used to study the influence of genetic variations on the circulating lipid responses promoted by certain nutrients or foods to provide specific dietary strategies considering the genetic factors in personalized nutrition interventions.

**Objective:**

To identify throughout a systematic review the potential nutrigenetic recommendations that demonstrate a strong interaction between gene-diet and circulating lipid variations.

**Methods:**

This systematic review used the PRISMA-Protocol for manuscript research and preparation using PubMed and ScienceDirect databases. Human studies published in English from January 2010 to December 2020 were included. The main results were outcomes related to gene-diet interactions and plasmatic lipids variation.

**Results:**

About 1,110 articles were identified, but only 38 were considered to fulfill the inclusion criteria established based on the reported data. The acquired information was organized based on gene-diet interaction with nutrients and components of the diet and dietary recommendation generated by each interaction: gene-diet interaction with dietary fats, carbohydrates or dietary fiber, gene-diet interaction with nutraceutical or dietary supplementation, and gene-diet interaction with proteins.

**Conclusion:**

Findings included in this systematic review indicated that a certain percentage of dietary macronutrients, the consumption of specific amounts of polyunsaturated or monounsaturated fatty acids, as well as the ingestion of nutraceuticals or dietary supplements could be considered as potential strategies for the development of a wide range of nutrigenetic interventions since they have a direct impact on the blood levels of lipids. In this way, specific recommendations were identified as potential tools in developing precision diets and highlighted the importance of personalized nutrition. These recommendations may serve as a possible strategy to implement as dietary tools for the preventive treatment and control alterations in lipid metabolism.

**Systematic Review Registration:**

https://www.crd.york.ac.uk/prospero/display_record.php?ID=CRD42021248816, identifier [CRD42021248816].

## Introduction

Overweight and obesity contribute to the development of various comorbidities associated with metabolic syndrome (MS), such as type 2 diabetes (T2D), hypertension, dyslipidemias, and cardiovascular diseases (CVD), which are the first causes of morbidity and mortality in the world (1). The pathogenesis of obesity is complex, and it involves environmental, sociocultural, physiological, medical, behavioral, genetic, epigenetic, and many other factors.

Although the main characteristic of obesity is the excess of adipose tissue, certain adipose tissue spots are associated with a higher risk of chronic diseases, such as that leading to increased waist circumference. Deposition of visceral fat leads to increased plasma levels of free-fatty acids and cytokines which can contribute to chronic inflammation, insulin resistance, and consequently, to the development of T2D, non-alcoholic fatty liver disease, dyslipidemias, and high blood pressure (2). Dyslipidemias comprise abnormally high concentrations and altered proportions of the different blood lipids and prelude to diverse CVD (3). Dyslipidemias are related to multiple factors such as genetic/hereditary (primary dyslipidemias) or an inadequate lifestyle (secondary dyslipidemias), which includes sedentary lifestyle, unbalanced diet, and excessive alcohol intake (4).

In this regard, it is emphasized that lifestyle habits, especially a healthy diet, are essential modifiable determinants for overweight and obesity, dyslipidemias, and CVD. However, genetic factors also play an important role in developing these pathologies (5). Individual responses to lifestyle modification vary, partially due to the genetic factor (6, 7). Data based on genome-wide association studies (GWAS) suggest that genotypic variations in specific nucleotides [single nucleotide polymorphisms (SNPs)] associated with obesity and dyslipidemias are factors of great importance behind this variable susceptibility (8). This has opened a new field for nutritional genomics around nutrigenetics, especially precision nutrition. These sciences have provided innovative evidence of the inter-individual interindividual variability and its relation with SNPs and nutrient intake since the human genome was completed (7). The postulates of nutrigenomics as applied science state that food components can act on the human genome, directly or indirectly, to alter the expression of genes and gene products. Diet can potentially compensate or accentuate the effects of genetic polymorphisms and the consequences of a particular diet depending on the balance of health, disease states, and an individual's genetic background (9).

Today's population nutrition guidelines are stratified to general characteristics (sex, age, or particular conditions like pregnancy). These recommendations are good for what they are made for, wide-reaching guidelines. Nonetheless, even with stratification for specific characteristics and conditions, they are not tailored to meet the needs of all individuals, especially for treating health conditions or reducing the risk of a disease integrating the genetic variants that confer susceptibility (10). A more exhaustive and individualized evaluation is required to include a variety of disease indicators, and therefore, a better disease stratification can be achieved (11). Thus, this systematic review aims to identify potential nutrigenetic recommendations that consider established interactions between gene-diet and blood lipid variations to provide possible strategies for personalized gene-diet treatment for dyslipidemias. However, it is crucial to consider that these critical points and potential nutrigenetic recommendations are subjects to specific populations and the results are reproducible in populations with the characteristics specified in each study.

## Methods

### Search Strategy for Systematic Review

This systematic review was developed according to the Preferred Reporting Items for Systematic Reviews and Meta-Analyses Protocol (PRISMA-P) guidelines (12). The review protocol was registered at PROSPERO under the CRD42021248816 registration number.

Studies published between January 2010 and December 2020, were identified by performing a comprehensive search in PubMed, the database of the National Center for Biotechnology Information (NCBI) of the US National Library of Medicine (US NLM), and ScienceDirect. In some cases, dietary recommendations were extracted directly from the original publications. The search strategy was composed of several MeSH terms (i.e., “Dyslipidemia” AND “polymorphism” AND “Diet,” “lipid polymorphism” AND “nutrients,” “Gene dietary recommendation” OR “genetic dietary recommendation”), which were revised and approved by all co-authoring researchers. Additionally, filters such as language restriction to English and studies that only involved humans, were applied. The detailed search strategies are available as Supplementary Table S1.

### Study Selection and Eligibility Criteria

Once titles and abstracts were filtered from the research databases, duplicate records were removed, the eligibility criteria were checked at three different stages, first applied only to titles, then after reading the abstracts, and once more by going through the full article. Inclusion criteria were: nutrigenetics, personalized diet, diet-gene interaction, mutation, genetics, genes, genomics, genotype, genetic variants, polymorphism, lipid alterations, lipid abnormalities, hyperlipidemia, dyslipidemia, cholesterol, hypercholesterolemia, triglycerides, hypertriglyceridemia, HDL-c, and LDL-c. This revision included studies conducted only in adults, randomized controlled trials, studies without randomization, crossover, cohort, case-control, and cross-sectional studies. Article abstracts were excluded if they included the following criteria: children or infants, maternal, pregnant women, elderly, reviews, meta-analysis, mental-related diseases, renal diseases, ocular diseases, respiratory diseases, familial dyslipidemias, hepatitis, dental diseases, inflammatory diseases, alcohol consumption, use of any drugs or medicine, or if the approached genes not directly related to lipid metabolism, did not measure lipid parameters, articles not focused on diet-gene interaction and lipid responses or if no statistically significant changes in the lipid profile were detected, either positive or negative changes.

### Data Collection Process

Two or three reviewers thoroughly analyzed all articles included in this review to determine eligibility. The most relevant information was captured in an electronic spreadsheet using the Population, Intervention, Comparison, Outcome (PICO) system (13). The data considered in the sample section were: sample size, ethnicity, age range, sex, physiological alterations or diseases of relevance to the study, allele frequency reported in the studies was also included. Regarding Intervention criteria, the following were considered: the administration of diets or structured feeding schemes, consumption of nutraceuticals or dietary supplements, and the application of Food Frequency Questionnaires (FFQ) or some other validated dietary assessment instrument to capture an individual's regular food consumption. A placebo, the consumption of a different diet or feeding scheme, the presence or absence of some polymorphism, and the genotype were considered as comparison criteria between treatments. The main results were outcomes related to gene-diet interactions and plasmatic lipids variation. Supplementary Table S2 shows the eligibility criteria of original studies in this review according to PICOS format.

### Quality Assessment and Risk Bias

The quality and the risk of bias for selected articles were assessed individually using either the JADAD Scale (14) for Randomized Control Trials, Crossover studies and interventional studies, or the Newcastle-Ottawa Scale (NOS) (15, 16) for cohort and cross-sectional studies. Both scales are procedures to assess the methodological quality of a clinical trial independently; JADAD evaluates a total of 11 quality items (maximum score: 13 points) and NOS 8 items (maximum score: 10 points). Criteria such as population selection, comparability between groups or treatments, exposure determination, clarity of methodology and results, and risk of bias are evaluated on both scales. The articles selected were those that obtained a quality assessment score greater or equal (≥) than 8 points on the Jadad scale (adequate quality) or 7 ≥ points according to NOS (good and excellent quality). In this manner, studies were categorized as excellent, good, and satisfactory quality, and records that failed the quality assessment were excluded. The risk of bias and quality assessment was performed by 2 reviewers and cross-checked to solve inconsistencies; in case of discrepancy, a third external reviewer evaluated the article. Besides, the level of evidence was categorized according to the US Preventive Service Task Force report (17).

### Data Analyses

The acquired information was organized based on gene-diet interaction with nutrients and components of the diet, as well as dietary recommendation generated by each interaction: gene-diet interaction with dietary fats (Table 1), gene-diet interaction with carbohydrates or dietary fiber (Table 2), gene-diet interaction with nutraceutical or dietary supplementation (Table 3) and, gene-diet interaction with proteins (Table 4).

**Table 1:** Gene diet interaction with dietary fats.

**Population**	**Gene variants and allele frequency**	**Intervention and comparison**	**Metabolic outcome**	**Nutrigenetic recommendation**	**Level of evidence and reference**
*n* = 424 MS RCT 12 months	*CETP* rs3764261 G = 77% T = 23%	Group 1: MedDiet rich in olive oil Group 2: LFD	T carriers displayed ↑ plasma HDL-c concentrations and ↓ TAG after MedDiet administration compared with GG homozygotes.	*CETP* rs3764261 TT/TG benefit from consuming a higher % of total dietary fat, especially a higher content of MUFAS obtained in the MedDiet patter to reduce TAG and increase HDL-c concentrations	I (18)
*n* = 553 37 **±** 8 year BMI > 28 kg/m^2^ Cross-sectional	*AGT*, rs699 C = 84% T = 16% *CETP*, rs5882 C = 50% T = 50% *CYP7A1*, rs1048943 A = 55% G = 45% *PPARG*, rs10856710 C = 55% G = 45% *APOB*, rs693 C = 82% T = 18% *APOE*, rs405509 T = 68% G = 32% *APOA1*, rs670 G = 83% A = 17% *ABCA1*, rs2230806 C = 65% T = 35% *LIPC*, rs1800588 T = 32% C = 68% *APOC3*, r5128 C = 56% G = 44%	Low fat intake vs. Hight fat intake	*CETP* rs5882 TT homozygotes showed: ↑ TC with a HFD. *PPARG2* rs10856710 C, *APOB* rs693 T and *APOE* rs405509 TT carriers showed: ↑ TC and LDL-c with a HFD, specifically, high in SFA. *APOA1* rs670 G carriers: associated with ↑ LDL-c with a HFD and a ↓ of HDL-c with a diet low in SFA *ABCA1* rs2230806 CC and *LIPC* rs1800588 TT homozygotes: associated to ↑ levels of TAG with a diet high in SFA *APOC3* r5128 C carriers: associated with ↑ levels of LDL-c with a HFD	*CETP* rs5882 TT and *APOC3* r5128 C carriers might benefit from a LFD to maintain healthy TC levels. *PPARG2* rs10856710 C, *APOB* rs693 T, *APOE* rs405509 TT, *APOA1* rs670 G, *ABCA1* rs2230806 CC and *LIPC* rs1800588 TT might benefit from a diet low in SFA to maintain healthy TC, LDL-c and HDL-c levels.	II-3 (19)
*n* = 120 21–60 year Moderate risk of CVD RCT 16 weeks	*APOE* rs405509 G = 52% T = 48% rs769450 G = 62% A = 38% rs439401 C = 64% T = 36% rs445925 G = 89% A = 11% rs405697 G = 75% A = 25% rs1160985 C = 54% T = 46% rs1064725 T = 95% G = 5% *LPL* rs320 T = 70% G = 30%	Group 1: HSFA diet Group 1: HMUFA diet Group 1: Diet high in *n*-6	*APOE* rs1064725, only TT homozygotes: ↓ TC after the HMUFA diet compared to the HSFA or *n*-6 PUFA diets.	*APOE* rs1064725 TT homozygotes benefit from higher intake of MUFAs in order to reduce TC levels.	I (20)
	rs328 C = 87% G = 13%				
*n* = 389 30–70 year risk of MS RCT 24 weeks	*APOE* rs429358 rs7412 E3/E3 = 56.6% E3/E4 = 25.7% E2/E3 = 13.8% E2/E2 = 0.8% E4/E4 = 1.3% E2/E4 = 1.8%	Group 1: Reference diet: HSFA/HGI Group 2: HMUFA Group 3: HMUFA Group 4: LF/HGI diet Group 5: LF/LGI diet	E4 carriers had greater ↓ in TC and apo B after LF/LGI diet. E4 carriers + HMUFA/HGI diet: ↓ TC to a lesser extent relative to E3/E3. E4 carriers + HMUFA/LGI diet: ↓ apo B to a lesser extent relative to E3/E3. E2 carriers + HMUFA/LGI diet associated with an ↑ of TAG.	*APOE* rs429358 and rs7412 E4 carriers are better responders to LF/LGI diet in order to have greater reductions in TC and apo B. E2 carriers benefit from a LFD in order to reduce TC.	I (21)
*n* = 1,466 > 18 year RCT 6 months	*APOE* rs429358 rs7412 E3/E3 = 62.9% E3/E4 = 22.5% E2/E3 = 10.4% E2/E2 = 0.4% E4/E4 = 2% E2/E4 = 1.8% E2 = 7% E3 = 79% E4 = 14%	Level 0: standard non personalized dietary and PA advice. Level 1: advice based on dietary intake and PA. Level 2: advice based on dietary intake, PA, and phenotype (blood biomarkers). Level 3: advice based on dietary intake, PA, phenotype, and genotype.	Gene-based personal Nutrition was associated with a smaller reduction in SFA intake than in non-gene based PN (level 2) for E4 carriers.	*APOE* E4 carriers benefit from a gene-based personalized nutrition to reduce SFA intake.	I (22)
*n* = 506 20–75 year Coronary heart disease RCT 3 year follow-up	*APOE* rs439401 C = 58.4% T = 41.6% rs440446 G = 55.75% C = 44.25% rs7412 C = 88.7% T = 11.3%	Group 1: MedDiet rich in EVOO Group 2: LFD	T carriers in the MedDiet group showed A ↓ in postprandial TAG compared with CC homozygotes.	It is recommended that *APOE* rs439401 T carriers include a diet rich in EVOO to decrease TAG levels.	I (23)
*n* = 88 51 **±** 9 year RCT 8 weeks	*APOE* rs7412 rs429358 E3/E3 = 50% E4/E3 = 50%	Group 1: LFD Group 2: HF/HSFA Group 3: HSF diet supplemented with 3.45 g DHA/d (HSF-DHA).	Genotype- diet interaction for TAG observed in *APOE* E3/E3 and *APOE* E3/E4 after the HSF-DHA diet relative to the LFD with 17% and 30% decreases	*APOE* E3/E3 and *APOE* E3/E4 may follow a HSF diet supplemented with 3.45 g DHA/d to reduce TAG levels.	I (24)
*n* = 200 18–25 year Cross-sectional	*APOA5* rs662799 T = 84% C = 16% rs3135506 C = 24% G = 76% *LEPR* rs8179183 G = 83% C = 17% rs1137101 A = 56% G = 44% *APOA2* rs3813627 GG = 100% rs5082	High SFA intake vs. Low SFA intake	*LEPR* rs1137101 AG/GG: ↑ risk of hypercholesterolemia. Also with an intake ≥12 g/day of SFA: 2.9 times ↑ risk of obesity and 2.4 times ↑ higher risk of hypertriglyceridemia than those with an intake <12 g/day SFA	*LEPR* rs1137101 AG/GG hetero and homozygotes might benefit from a low SFA intake (<12 g/day SFA)	II-3 (25)
	T = 85% C = 15%				
*n* = 734 30–70 year Overweight/obese RCT	*APOA5* rs964184 C = 16% G = 84%	Group 1: LF/HCD Group 2: LF/HPD Group 3: HF/LCD Group 4: HF/HPD	G carriers of the LF groups: ↓ in TC and LDL-c than non-carriers. G carriers of the HF groups: ↑ in HDL-c than non-carriers	*APOA5* rs964184 CG and GG benefit from a LFD to reduce TC and LDL-c, however, a HFD is needed to increase HDL-c levels.	I (26)
*n* = 1,465 20–65 year BMI > 26 kg/m^2^ Cross-sectional	*APOA5* rs662799 C = 6% T = 94%	High SFA intake vs. Low SFA intake	In C carriers fat intake was inversely related to TAG levels.	*APOA5* rs662799 CC/CT benefit from a LFD to control TAG levels.	II-3 (27)
*n* = 282 20–65 year BMI > 30 RCT 12 weeks	*APOA1* rs670 G = 81% A = 19%	Group 1: HFD Group 2: LFD	A carriers showed ↑ HDL-c levels with LFD.	*APOA1* rs670 A carriers benefit from a LFD to elevate HDL-c levels.	I (28)
*n* = 549 30–70 year RCT 24 weeks	*PPARG* rs1801282 C = 89% G = 11%	Group 1: Reference diet HSFA Group 2: HMUFA diet Group 3: LFD	Low dietary PUFAS:SAT ratio: ↑ TC and LDL-c in G carriers (Ala12) than in noncarriers.	*PPARG* rs1801282 G (Ala12) carriers should maintain high dietary PUFAS:SAT ratio (>0.65) to improve TC and LDL-c levels.	I (29)
*n* = 466 30–70 year MS RCT	*PPARG* rs1891282 C = 90% G = 10% *PPARA* rs1800206 L = 94% V = 6%	Group 1: Reference diet HSFA Group 2: HMUFA diet Group 3: LFD	Co-carriers of the *PPARG*-G (Ala12) and *PPARA* V (Val162) had ↓ LDL-c and ↓ sdLDL after the HMUFA diet	Co-carriers of the *PPARG*-G (Ala12) and *PPARA* V (Val162) benefit from a HMUFA diet to lower LDL-c and sdLDL levels.	I (30)
*n* = 743 30–70 year OW and OB RCT 2 years	*LIPC* rs2070895 A = 26% G = 74%	Group 1: LF/LPD Group 2: LF/HPD Group 3: HF/LPD Group 4: HF/HPD 750-kcal/d deficit from baseline and 20- g/day dietary fiber all groups	LFD: A carriers were associated with ↓ of TC and LDL-c concentrations, whereas an opposite genetic effect was found in the HFD group. LFD: Significant genotype-time interactions on changes in TC, LDL-c, and HDL-c	*LIPC* rs2070895 A carriers benefit from a LFD diet to improve TC and LDL-c levels.	I (31)
*n* = 41 20–64 year RCT Crossover	*LIPC* rs1800588 T = 40% C = 60%	Group 1: High fat “Western diet” (39% fat) Group 2: Low fat “Hispanic” traditional diet (20% fat)	CC/CT homo and heterozygotes showed: ↑ HDL-c and ↑ LDL-c levels, following the western diet compared to the Hispanic diet.	*LIPC* rs1800588 CC/CT might benefit from a HFD (39% total fat) to raise HDL-c levels but may also raise LDL-c levels.	I (32)
*n* = 6,880 55–80 year T2D or 3 or more CV risk factors RCT 4.8 years	*LPL* rs13702 T = 67% C = 33%	Group 1: MedDiet + EVOO (30 g/day) Group 2: MedDiet + nuts (30 g/day) Group 3: Control group (LFD)	*LPL* rs13702 associated with ↓ TAG in C carriers By the 3rd year, C carriers still showed ↓ in TAG with MedDiet, high in PUFAS and MUFAS	*LPL* rs13702 C carriers benefit from a high-unsaturated fat MedDiet intervention (30 g/day EVOO) to reduce TAG.	I (33)
*n* = 109 18–65 year OW and OB RCT 8 weeks	*FABP2* rs1799883 G = 72% T = 28%	Group 1: Moderate fat diet intake	GG (Ala54Ala) and GT (Ala54Thr) benefited equally in the reduction of TAG, TC and VLDL-c after diet, compared with baseline results	*FABP2* rs1799883 GG and GT benefit equally from the reduction of TAG, TC, and VLDL-c, after ingesting a moderate-fat diet (Fat: 30%, Protein: 15% CHO: 55%. Fiber: 25 g/day, plant stanols/sterols: 2 g/day)	I (34)
*n* = 111 OB RCT 3 months	*FABP2* rs1799883 G = 51% T = 49%	High PUFA hypocaloric diet.	T carriers (Thr54) had better metabolic response after a high PUFA hypocaloric diet than obese patients with GG (Ala54Ala) homozygotes with ↓ TC and LDL-c	*FABP2* rs1799883 T carriers benefit from consuming a High PUFA diet in order to decrease TC and LDL levels	I (35)
*n* = 200 > 18 year Vegetarians Cross-sectional	*FADS1* rs174547 C = 45% T = 55%	LA and ALA intake (g/day): LA Low (≤ 5.8) LA Medium (5.8–8.1) LA High (≥ 8.2) ALA Low (≤ 0.45) ALA Medium (0.4–0.6) ALA High (≥0.65)	Vegetarians TT homozygotes with medium LA and medium ALA intake had ↓ HDL-c levels.	The dietary intake of LA may interact with rs174547 in *FADS1* gene to affect HDL-c level especially among vegetarians TT homozygotes, which warrants the needs to monitor the amount of dietary LA intakes in vegetarians' daily diet.	II-3 (36)
*n* = 3,575 46.7 ± 9.8 year Cohort	*FADS1* rs174546 T = 33% C = 67% *FADS2* rs174570 T = 15% C = 85% rs482548 T = 9% C = 91%	Group 1: *n*-3 PUFAs (low: <0.51 % of energy) *n*-3 PUFAs (high > 0.51 % of energy) Group 2: *n*-6 PUFAs (low, <5.26% of energy) *n*-6 PUFAs (high: >5.26 % of energy).	*FADS1* rs174546 C carriers: ↓ TC, and ↑ HDL levels with a High intake of *n*-3 PUFAs. *FADS1* rs174546, C carriers showed : ↑ HDL-c concentrations in the group with a High intake of *n*-6 PUFAs but not in the group with a low intake.	*FADS1* rs174546 C carriers increase HDL-c levels with either a high intake of *n*-3 (0.51 % of energy) or *n*-6 (>5.26 % of energy)	II-2 (37)
*n* = 592 39.3 ± 16.0 year OB Cross-sectional	*FASN* rs4246444 A = 38% C = 62%	3-day regular diet dietary record Group 1: HFD Group 2: LFD	*FASN* rs4246444 A carriers with high fat consumption present a ↓ on LDL-PPD and increase of number of small, dense LDL particles as well as the cholesterol levels contained in the small LDL fraction	*FASN* rs4246444 A carriers might benefit from a LFD to avoid LDL-c PPD increase and number of LDL-c particles.	II-3 (38)
*n* = 507 20–75 year MS RCT 12 months	*TNF-α* rs1800629 G = 85% A = 15%	Group 1: MedDiet Group 2: LFD (AHA and NCEP dietary guidelines)	*TNF-α* rs1800629 GG homozygotes: ↓ TAG levels following a MedDiet	*TNF-α* rs1800629 GG benefit from a MedDiet to reduce TSG levels.	I (23)
*n* = 261 BMI > 30 kg/m^2^ RCT 3 months	*TNF-α* rs1800629 G = 87% A = 13%	Group 1: HMUFAS diet (30–40 ml/d EVOO and 40–50 g/day of walnuts) Group 2: HPUFAS diet (30–40 ml/d of sunflower oil and 3 servings of oily fish/week)	*TNF-α* rs1800629 G carriers: ↓ TC, LDL-c and TAG levels after High PUFAS diet	A high PUFA hypocaloric diet benefits *TNF-α* G carriers to reduce TC, LDL-c and TAG levels.	I (39)
*n* = 7,166 55–80 year High CV risk RCT	*MLXIPL* rs3812316 C = 91% G = 9%	Group 1: MedDiet with EVOO (4 tbsp/d) Group 2: MedDiet with nuts Group 3: LFD.	G carriers: ↓ TAG concentrations in the MedDiet vs. LDF	*MLXIPL* rs3812316 G carriers benefited from MedDiet eating pattern to reduce risk of hypertriglyceridemia.	I (40)
*n* = 248 43.2 ± 14.1 year OB RCT 3 months	*FAAH* rs324420 C = 84% A = 16%	Group 1: LFD Group 2: LCD	CC homozygotes showed: ↓ TC and TAG after the LFD administration. CA or AA showed: ↓ only in TC levels after LFD	*FAAH* rs324420CC homozygotes may benefit from the consumption of either a LFD to reduce TC and TAG or a LCD in order to decrease TC and LDL-c levels *FAAH* rs32442 A carriers may benefit from the consumption of a LFD, in order to decrease only TC levels	I (41)
*n* = 56 22.89 ± 1.80 year RCT *Crossover*	*SREBP-1c* rs2297508 G = 84% C = 16%	Wash-out diet for 7 days, followed by the HC/LFD for 6 days.	*SREBP*-1c female C carriers: ↓ TAG with HC/LFD intake and e TAG in female subjects	A HC/LFD is associated with a delayed increase in serum TAG of *SREBP-1c*, rs2297508 C female carriers and with elevated serum HDL-c in C male carriers.	I (42)

***↑**, high/higher; **↓**, low/lower/decrease; CV, cardiovascular; EVOO, extra virgin olive oil; HCD, high carbohydrate diet; HCCD, high-complex carbohydrate diet; HDL-c, high density lipoprotein; HFD, high fat diet; HSFD, high saturated fat diet; HGID, high glycemic index; HMUFAD, high-monoinsaturated fat diet; HPD, high protein diet; HPUFAD, high-polyunsaturated fat diet; FFQ, Food Frequency questionnaire; LCD, low carbohydrate diet; LDL-c, Low density lipoprotein; LFD, Low fat diet; LGID, low glycemic index; LPD, low protein diet; MedDiet, mediterranean diet; MS, metabolic syndrome; OB, obese; OW, Overweight; PA, physical activity; P:S, polyinsaturated:saturated ratio; RCT, randomized control trial; sdLDL, small dense LDL; TC, total cholesterol; TAG, triglycerides; T2D, type 2 diabetes*.

**Table 2:** Gene diet interaction with carbohydrates and fiber.

**Population**	**Gene variants and allele frequency**	**Intervention and comparison**	**Metabolic outcome**	**Nutrigenetic recommendation**	**Level of evidence and reference**
*n* = 30 27–78 y. RCT crossover	*CYP7A1* rs3808607 T = 55% G = 45%	Diet 1: 3 g High molecular weight (HMW) β-glucan/d Diet 2: 3 g Low molecular (LMW)weight β-glucan/d Diet 3: 5 g Low molecular weight β-glucan/d Control Diet: Wheat and rice (WR)	*CYP7A1* rs3808607 G carriers showed greater responses to 3 g HMW β-glucan/d in lowering than TT homozygotes.	*CYP7A1* rs3808607-G carriers (GG or GT) might benefit the most with 3 g HMWβ-glucan/d intake than TT carriers to reduce TC and LDL-c levels.	I (43)
*n* = 82 > 21 y. RCT crossover 8-weeks	*CETP* rs708272 G = 59% A = 41%	Group 1: Two green kiwifruits/d + healthy diet Group 2: healthy diet alone	At baseline, GG (B1/B1) homozygotes: ↑ TC:HDL-c and TAG:HDL-c ratio, than A (B2) carriers. After kiwifruit intervention: G (B1) carriers showed greater ↓ TAG:HDL-c than healthy diet alone	It is recommended to include 2 kiwi fruits/d to *CETP* rs708272 GG (B1/B1) homozygotes to improve TAG:HDL-c ratio.	I (44)
*n* = 641 19.1 ± 2.3 y. Cohort	*AHSG* rs2518136 C = 46% T = 54% rs4917 T = 42% C = 58%	Macronutrient intake	Greater CHO intake, especially higher sucrose intake and lower SAT intake were associated with elevated circulating TAG. Associations were stronger in *AHSG* rs4917 CT heterozygotes.	*AHSG* rs4917 is associated with higher levels of TAG with a higher carbohydrate intake, especially, a high sucrose intake and low in SFA intake.	II-2 (45)
*n* = 283 40–50 y. RCT	*APOA5* rs662799 T = 64.9% C = 35.1%	DIRE program: Replace 1/3 of refined rice intake with legumes 3 times/d, increase vegetable intake to at least 6 units/d +30-min walk after dinner each day	TT homozygotes had greater ↓ of TAG and ↑ HDL-c levels	It is recommended that *APOA5* rs662799 TT increase intake of non-refined carbohydrates, 6 units of vegetables (30–60 g each) to reduce TAG and elevate HDL-c	I (46)
*n* = 56 Healthy youth 22.89 ± 1.80 y. Crossover	*APOC3* rs5128 C = 75% G = 25%	Wash-out diet for 7 days, followed by the HCD//LFD for 6 days.	G (S2) carriers: ↑ TAG: HDL-c ratio than CC (S1S1) homozygotes after the HCD. TC: HDL-c and LDL-c:HDL-c ratios: ↓ after the HCD regardless of genotype. Female G carriers: ↑ TAG: HDL-c ratio than CC homozygotes females before or after the HCD.	A HCD/LFD promotes the reduction of TC:HDL and LDL: HDL ratios regardless of gender and of genotype of the *APOC3* rs5128 polymorphism.	II-1 (47)

*BMI, Body Mass Index; HCD, high carbohydrate diet; HDL-c, high density lipoprotein; HMW, high molecular weight; LDL-c, low density lipoprotein; LFD, low fat diet; LGID, low glycemic index; LMW, low molecular weight; RCT, randomized control trial; SFA, saturated fatty acid; TC, total cholesterol; TAG, triglycerides; ↑: high/higher; ↓: low/lower/decrease*.

**Table 3:** Gene variants and nutraceutical or dietary supplementation.

**Population**	**Gene variants and allele frequency**	**Intervention and comparison**	**Metabolic outcome**	**Nutrigenetic recommendation**	**Level of evidence and reference**
*n* = 43 47.22 ± 6.5 y. Dyslipidemia RCT 6 weeks	*MTHFR* rs1801133 Group 1: C = 66% T = 34% Group 2: C = 55% T = 45%	Group 1: Watermelon extract (*Citrullus lanatus*): 6 g/day Group 2: Placebo: 6 g/day (mixture of sucrose/glucose/fructose)	Watermelon extract: ↓ plasma TC, and LDL-c levels in T carriers of group 1. Plasma TAG ↑ in group 2.	*MTHFR* rs1801133 T carriers benefit the most from a Watermelon extract (6 g/day) administration to reduce LDL-c.	I (48)
*n* = 60 19–65 y. OB/OW RCT 12 weeks	*PPARcy2* rs3856806 C = 84% T = 16%	Group 1: *Kochujang* (32 g/day: 39 g/day) Group 2: Placebo	C carriers: ↓ TAG levels and TAG: HDL-c ratio after Kochujang intake.	*PPARcy2* rs3856806 C carriers benefit from *Kochujang* (32 g/day) supplementation to reduce TAG	I (49)
*n* = 4,262 men and 4,813 women. 30–70 y. Cross-sectional	*LIPC* rs1800588 Men C = 65% T = 35% Women C = 64% T = 36% *CEPT* rs1800775 Men A = 50% C = 50% Women A = 51% C = 49%	Coffee drinkers (drank coffee > 3/week) Non-drinkers coffee	Significant interaction of *LIPC* TT Women—coffee consumption—higher HDL-c levels. *CETP* CC homozygotes: significantly associated with lower levels of HDL-c.	*LIPC* rs1800588 TT homozygotes that drank coffee three or more times per week were associated with higher levels of HDL-c	II-3 (50)
*n* = 150 35–85 y. T2D RCT 180 days	*CD36* rs1527483 G = 78% A = 22% *NOS3* rs1799983 C = 93% A = 7% *PPARG* rs1801282 C = 94% G = 6%	Omega-3 fatty acid groups Fish oil group: 2 g/day. Flaxseed oil group: 2.5 g/day. Control group: corn oil	*CD36* GG homozygotes: ↓ TAG after fish oil supplements. *PPARG* interaction: ↓ LDL-c levels after Omega-3 supplements *NOS3:* Omega-3 supplements interacted with genotype by ↓ TAG, TC, TC:HDL-c ratio	Subjects with T2D, carriers of *CD36*-G *PPARG*-G and *NOS3*-A allele tended to respond better to *n*-3 fatty acids in improving lipid profiles (TG, LDL-c, and LDL-c TC, respectively) This interaction is mainly attributed to the fish oil supplements (2 g/day of C20:5n-3 and C22:6n-3)	I (51)
*n* = 38 18–70 y. RCT crossover	*APOE* rs429358 rs7412 E3/E3 and E3/E4 E3/E3 = 53% E3/E4 = 47%	Control oil: (80:20 palm olein: soyabean mixture) EPA-rich oil (ERO, 3.3 g EPA/day) DHA- rich oil (DRO 3.7 g DHA/day)	Participants of all 3 groups: showed ↓ TAG levels. E3/E4 with ERO intervention: ↓ 5.4% in TC relative to baseline. E3/E4 with DRO treatment: ↓ LDL-c over the 4-week intervention period, with a non-significant decrease in the E3/E3.	*APOE* E3/E4 benefit from DHA supplementation (3.3–3.7 g/day) to reduce TAG. Monitoring DRO supplementation among E3/E4 carriers is recommended.	I (52)
*n* = 71 30–75 y. RCT crossover 28 days	*APOE* rs429358, rs7412 E3/E4 E3 = 63% E4 = 37% *CYP7A1* rs3808607 T = 60% G = 40% *CETP* rs5882 NR *ABCG8* rs4148217 NR	Individuals classified as High or Low endogenous cholesterol synthesis (HECS/LS) Period 1: consumption of habitual diet. Plant Sterols period: consumed 2 g PS/d	HECS individuals did not show LDL-c lowering whereas individuals with LS showed reductions *CYP7A1* rs3808607 GT/GG and *APOE* E4 (especially) associated with LDL-c lowering in response to PS consumption.	*APOE* E3 and E4 carriers and *CYP7A1* rs3808607 GT/GG benefit from a 2 g plant sterol supplementation to reduce LDL-c levels.	I (53)
*n* = 5,576 Cross-sectional	23 SNPs including: *ABCA1* rs4149268, rs3890182	Data for dietary intake were collected through a 24-h dietary recall.	The association between LDL-c and *APOB* rs693 T allele and vitamin E in Mexican Americans was the most significant. The *PCSK9* rs11206510 x	Vitamin E may modify *APOB* rs693 lipid trait genetics in Mexican American population.	II-3 (54)
	*CETP* rs9989419, rs3764261 *APOB* rs693, rs754523 *LIPC* rs3890182 *LPL* rs2197089 NR		vitamin A interaction was associated with LDL-c in Mexican Americans		

***↑**,*High/Higher;* **↓**, low/lower/decrease BMI, Body Mass Index; DHA, docosahexaenoic acid; EPA, eicosapentaenoic acid; HDL-c, high density lipoprotein; HECS, high endogenous cholesterol synthesis; HPD, high protein diet; LDL-c, low density lipoprotein; LCD, low carbohydrate diet; LECS, low endogenous cholesterol synthesis; Marine n-3, C20:5n-3, C22:5n-3, C22:6n-3; MUFAs, monounsaturated fatty acids; MedDiet, Mediterranean diet; MS, metabolic syndrome; NR, not reported; n-3, Omega 3; n-6, Omega 6; Plant based n-3, C18:3n-3; PUFAs, polyunsaturated fatty acids; RCT, randomized control trial; SFA, saturated fatty acid; TC, total cholesterol; TAG, triglycerides; T2D, type 2 diabetes*.

**Table 4:** Gene variants interaction with dietary protein.

**Population**	**Gene variants and allele frequency**	**Intervention and comparison**	**Metabolic outcome**	**Recommendation**	**Level of evidence and reference**
*n* = 193 BMI > 30 RCT 9 months	*FABP2* rs1799883 Group 1 G = 63% T = 37% Group 2 G = 70% T = 30%	Group 1: HPD/LCD Group 2: Standard Hypocaloric Diet	With both diets and only in GG homozygotes, TC and LDL-c levels decreased	*FABP2* G (Ala) carriers benefit equally from a hypocaloric diet either HPD/LCD or standard diet to reduce TC and LDL-c levels.	I (55)
*n* = 283 OB RCT 9 months	*UCP3* rs1800849 Group 1 C = 71.% T = 29% Group 2 C = 67 % T = 33%	Group 1: HPD/LCD Group 2: Standard Hypocaloric Diet	With both diets and only in T/T (wild genotype), TC, LDL-c decreased. The improvement in these parameters was similar in both groups	*UCP3* rs1800849 CC carriers benefit from a hypocaloric diet (<1,093 kcal/day) (either HPD/LCD or standard) to improve TC and LDL-c levels.	I (56)

*BMI, Body Mass Index; HPD, high protein diet; HDL-c, high density lipoprotein; LCD, low carbohydrate diet; LDL-c, low density lipoprotein; MUFAs, monounsaturated fatty acids; PUFAs, polyunsaturated fatty acids; RCT, randomized control trial; TC, total cholesterol; TAG, triglycerides*.

## Results

### Study Selection and Characteristics

**Figure 1:**
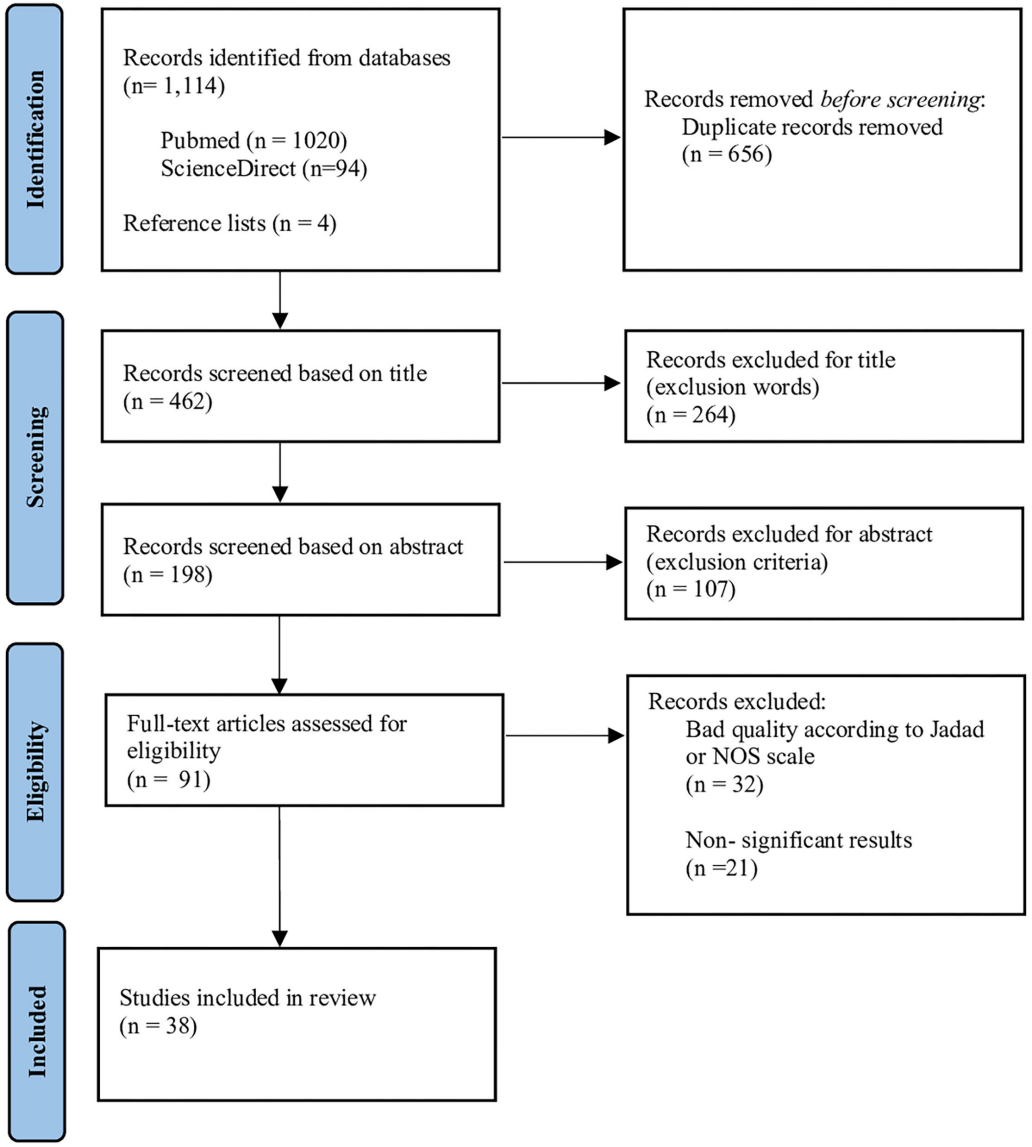
PRISMA flow diagram summarizing the selection process of the included studies.

Figure 1 summarizes the search and study selection process using the PRISMA flow diagram. An initial sample of 1,118 articles was identified, 1,114 from databases, and 4 articles by reference lists. 656 duplicates were subsequently excluded, the remaining 462 articles were screened for title and abstract, based on exclusion word and criteria, previously mentioned, resulting in the removal of 371 articles. Then, 91 articles were full-text analyzed. A total of 32 articles were excluded, i.e., 21 articles did not present significant results in terms of a decrease in lipid parameters after treatment or no gene-diet interaction identified, and 32 articles were excluded because the methodologies were recognized as weak, according to quality assessment scales (NOS and Jadad). Finally, 38 articles were selected for the systematic review, of which 29 were randomized controlled trials (RCT), 2 cohorts and 7 cross-sectional studies. The quality and the level of evidence of these included articles are provided in detail in Supplementary Table S3. Results showed that 29 RCT evaluated with the Jadad scale had adequate quality (score ≥ 8 points) and level I of evidence (AHRQ scores). Regarding the items evaluated by the scale, only 5 double-blinded studies were identified; the rest were single-blinded and unblinded. All articles clearly defined the objectives of the study, the inclusion and exclusion criteria, and the statistical methods used. All studies had at least one control group or comparison group, and it is highlighted that 24 articles present in their methodology the justification of the sample size used (statistical power calculation).

Cohort and cross-sectional studies evaluated with the Newcastle-Ottawa Scale (NOS) had scores ranging from 7 to 9 (good and excellent quality). It should be noted that all the articles fulfilled more than 98% of all items evaluated by the NOS scale, such as satisfactory representativeness of the sample, justification or the sample size, exposure verification, comparability between subjects from different groups, evaluation of the results and precise description of the statistical tests used. See Supplementary Tables S4, S5 for detailed information.

The included studies showed significant heterogeneity regarding sample size, population characteristics, type of dietary intervention, genes and polymorphisms evaluated, allele frequency, and experimental analyses. Most of the studies included derived from major clinical trials like PREDIMED (Prevención con Dieta Mediterránea), CORDIOPREV (CORonary Diet Intervention with Olive oil and cardiovascular PREVention study), RISCK, POUNDS lost, and Food4Me. All of the studies were performed in adults (19–85 y/o) males or females who met all inclusion criteria, with a total sample size of *n* = 47,956 and an average sample size of *n* = 1,262 for all 38 studies included.

A large part of the population evaluated in the included studies were from Spain (*n* = 11) and China (*n* = 6), and a majority of the studies included Caucasians since they were carried out in countries such as the United Kingdom (*n* = 3), the United States (*n* = 3), Canada (*n* = 2), and other countries such as The Netherlands and New Zealand (*n* = 5). The rest of the studies included populations from Mexico, Brazil, Korea, and the Inuit population. The list of genes that predominated in this review included: *CETP, AGT, CYP7A1, PPAR, APOB, APOE, APOA, APOC, LPL, ABCA1, LIPC, FADS1, FASN, FAAH*, and *SREBP-1C*. There are several genes whose participation indirectly alters lipid metabolism *(FTO, GHRELIN, LEP, POMC*, among others); their influence is significant and remarkable, but this review considered genes directly involved in lipid metabolism.

Gene-diet interaction with nutrients or diet components identified in included articles were varied but were clearly categorized into four major groups, developed as main points throughout this review: studies that approached dietary fat intake recommendations and gene variants (*n* = 26), studies that addressed dietary recommendations related to gene variants and carbohydrate or dietary fiber intake (*n* = 6), dietary recommendations related to gene variants and nutraceuticals and dietary supplements intake (*n* = 7) and studies about dietary recommendations related to gene variants and protein intake (*n* = 2).

#### Dietary Fat Intake Recommendations and Gene Variants Related to Fat

The official recommendation for achieving dietary fat for the healthy adult population is 20–35% of total calorie intake. However, it is equally important to emphasize the quality of fats in this percentage. A low-fat diet (LFD) could be considered when fat provides <20% total calorie intake, a moderate-fat diet 20–35%, and a high-fat diet (HFD) > 35% (57). Convincing evidence has shown that maintaining a healthy unsaturated/saturated fat balance might be more beneficial for reducing the risk of chronic diseases (58). Nevertheless, these recommendations do not consider the genetic background of different individuals. The findings regarding the interaction between gene variants and dietary fat intake are broken down below (Table 1).

##### Cholesteryl Ester Transfer Protein

*CETP* is a protein synthesized in the liver, and its function is to mediate the transfer of cholesterol esters from HDL (High-density lipoprotein) to VLDL (Very low-density lipoprotein), chylomicrons, LDL (Low-density lipoprotein), and the transfer of triacylglycerols (TAG) from VLDL and chylomicrons to HDL. When this protein is inhibited, increased HDL-c and decreased LDL-c occur (59).

With attention to the *CETP* gene, Garcia-Rios et al. (18) evaluated 424 adults with metabolic syndrome in an RCT that compared the Mediterranean diet (MedDiet) with a low-fat diet (LFD). *CETP* rs3764261 T-carriers (TT and TG) displayed higher HDL-c plasma concentrations (*p* = 0.021). TT and TG had lower TAG concentrations following the same diet than GG (*p* = 0.020). In this manner, the final recommendation for *CETP* rs376461 TT and TG is to increase the intake of monounsaturated (MUFAs) (22%) and polyunsaturated fats (PUFAs) (6%) and reduce intake of saturated fats (SFA) (>7%).

On the other hand, Rudkowska et al. (19) also evaluated *CETP* polymorphism in a cohort study in an Inuit population where *CETP* Ile405Val rs5882 TT was associated with elevated TC in subjects with a high fat intake (*p* = 0.0460). Therefore, this genotype might benefit from an LFD (19). It is essential to state that this recommendation might be specific only for the Inuit population.

##### Apolipoprotein E

*APOE* is perhaps one of the most studied genes for gene-diet interactions due to its importance as an essential regulatory protein in cholesterol homeostasis. It is a transporter for TAG-rich lipoproteins and a major ligand for transport to the liver by receptor-mediated endocytosis (21). *APOE* is a crucial component of plasma lipoproteins because it is involved in their production, conversion, and clearance. Specifically, *APOE* associates with chylomicrons and its remnants, VLDL, IDL, and shows preferential binding to HDL-c. It also binds with other receptors like the LDL and VLDL receptors. It is estimated that *APOE* E3/E4 and E4/E4 variants are associated with higher risks (22 and 45%, respectively for developing cardiovascular disease than E3/E3) (60).

Shatwan et al. (20) studied various *APOE* polymorphisms. They found a specific gene-diet interaction with *APOE* rs1064725 TT homozygotes, as they had significantly lower total cholesterol (TC) concentrations after a high MUFA (monounsaturated fatty acids) diet compared with a high SFA (saturated fatty acids) diet and ω-6 PUFA (polyunsaturated fatty acids) diet (*p* = 0.003 and *p* = 0.004). According to the authors, a possible explanation for this gene-diet interaction is that a high MUFA diet could shorten the residence time of very-low-density lipoprotein (VLDL) particles in the circulation, therefore, regulating and increasing TAG-rich lipoproteins clearance rate among TT homozygotes of *APOE* rs1064725.

21 investigated the effect of replacing saturated fatty acids (SFA) with either MUFAs or carbohydrates of high or low glycemic index (GI) in five different possible pattern combinations of *APOE* rs429358 and rs7412. After 24 weeks of dietary intervention, the five patterns resulted in different significant interactions between E2, E3/E3, and E4 carriers; *APOE* E3/E3 carriers benefit from a high MUFA/high GI diet in order to reduce TC (*p* = 0.03); E4 carriers benefit the most from a low fat/low GI diet (*p* = 0.03), and E2 carriers benefit from an LFD to maintain healthy circulating levels of TAG (*p* = 0.02). The article mentions that the isoforms of *APOE* alter the physical structure of the lipoprotein itself. E2 isoform, for example, reduces the affinity of the particle to bind to LDL receptors. This difference explains why E2 transport is associated with low levels of LDL-c.

On the other hand, there may be less competition in uptake for the E2 isoform and its content. The association of E4 carriers with dyslipidemia is not so clear; this isoform is preferentially incorporated into VLDL, and this could compete with the uptake of LDL by the LDL receptor, thus decreasing the hepatic LDL uptake and prolonging its presence in circulation. This mechanism is supported by other different *in vitro* studies and may explain why E4 carriers express a greater sensitivity to dietary fat reduction, specifically SFAs (20, 61).

Fallaize et al. (22) studied *APOE* rs429358 and rs7412 in an RCT and found that through different levels of gene-personal nutrition. Gene-based personalized nutrition was associated with a significant reduction in total fat and SFA intake compared to a not gene-based personalized nutrition approach in E4 carriers. There was also a significant impact of genotype on the change in TC concentrations after dietary advice intervention (total fat, *p* = 0.016; SFAs, *p* = 0.025; MUFAs, *p* = 0.019; PUFAs, *p* = 0.024). The study advocates for dietary information based on personal genetics as much more understandable and valuable than general dietary guidelines and suggests that it could help to increase the motivation for effective dietary changes. Nevertheless, this finding could be exclusive to this study and does not recount to personalized recommendations for individual health benefits.

In this context, Gomez-Delgado et al. (61) evaluated patients with coronary heart disease from the CORDIOPREV RCT study, which compared the MedDiet (high in MUFAs) and an LFD (<30%). After 3 years, T carriers in the MedDiet group had a significantly larger reduction in postprandial TAG levels than CC (homozygous wild type) (*p* = 0.03). So, it is recommended that *APOE* rs439401 T carriers follow a MedDiet pattern to decrease TAG levels. These results add up to a final recommendation for *APOE* rs439401 and rs7412 benefit from the MedDiet pattern with a moderate fat intake high in MUFAs and low in SFA. This article provides an explanation for these findings based on the fact that a diet rich in MUFAs increases the secretion of VLDL-c (that contains *APOE)* and IDL-c, leading to faster elimination of TAG from the bloodstream, thus preventing its conversion to LDL-c and, possibly, reducing its atherogenic effect.

Carvalho-Wells et al. (24) found that *APOE* rs7412 and rs429358 E4/E4 and E3/E4 experienced more significant reductions in TAG with a high SFA diet and 3.45 g ω-3 supplementation, than with the LFD (*p* = 0.033). These findings can be interpreted as an additional therapeutic effect of ω-3 for these polymorphisms.

##### Apolipoprotein A1

*APOA1* gene encodes a protein that plays a fundamental role as a component of HDL. It plays a pivotal role in reverse cholesterol transport as it promotes cholesterol transportation from tissues to the liver for excretion. It is also a cofactor for cholesterol acyltransferase (LCAT), the enzyme responsible for most plasma cholesteryl esters' formation (62).

De Luis et al. (28) evaluated 282 adults with obesity in an RCT for 12 weeks and analyzed *APOA1* rs670. All participants were randomized into an LFD (27% fats) or an HFD (38%), both diets with a caloric restriction of 500 kcal based on individual estimates. A carriers with LFD showed increased levels of HDL-c after the LFD (*p* = 0.03). It is essential to mention that A carriers already had elevated HDL-c before the dietary intervention; previous studies had made similar observations. On the other hand, Rudkowska et al. (19) reported that *APOA1* rs670 G in an Inuit population cohort study is associated with an increase in LDL-c with an HFD, yet decreased levels of HDL-c were noticed with a diet low in saturated fat. These two studies support the recommendation for *APOA1* rs670 to follow an LFD. However, both studies share the limitation of not specifying the type of unsaturated fats used in the intervention, such as MUFAs and PUFAs. They only refer to the total percentage of fat, so the potential interactions between these types of fat may have been overlooked.

##### Apolipoprotein A5

Apolipoprotein A5 (*APOA5*) is produced in the liver, and its primary lipoprotein associations are VLDL-c, chylomicrons, and HDL-c. Its primary function is promoting lipoprotein lipase (LPL) activity on TAG lipolysis (59). For this reason, *APOA5* plays a pivotal role in regulating plasma TAG, a risk factor for cardiovascular disease (63). Genetic studies have reported that polymorphisms in *APOA5* are among the most determinant factors for developing elevated TAG levels. *APOA5* CC homozygotes and TC heterozygotes have been associated with increased cardiovascular disease in various populations (64).

Zhang et al. (26) studied 734 overweight or obese adults who were randomly assigned to four different diets with different percentages of energy derived from fat, protein, and carbohydrate, and found that *APOA5* rs964184 CG and GG benefit from LFD (<20% energy from fat) to reduce TC and LDL-c. This is concurrent with Sánchez-Moreno et al. (27) found with *APOA5* rs662799 in a cross-sectional study in adults with a BMI > 26 kg/m^2^, where TC heterozygotes seemed to benefit from an LFD for controlling VLDL and TAG levels. These coincident results and recommendations suggest that this gene-diet interaction could be a trend in obese individuals; however, it is also possible that an impaired efficiency of ribosomal translation in the −1131C allele may lead to a reduced LPL-mediated TAG uptake into adipocytes. Another possible mechanism is linked to the regulation of *APOA5* by thyroid hormones, or peroxisome proliferator-activated receptors (*PPARs)* (20, 65).

##### Peroxisome Proliferator-Activated Receptors

The *PPAR* family encompasses critical transcriptional regulators of lipid and carbohydrate metabolism. The isotypes of *PPAR* include *PPAR*α, *PPAR*δ, and *PPAR*γ, and these codify a great variety of genes responsible for glucose and carbohydrate homeostasis as well as cholesterol metabolism. Any alteration in these genes can lead to obesity and cardiovascular disease. For example, the C allele of *PPAR*α is a crucial regulator of lipid metabolism and has been known to have cardioprotective effects because it associates with higher levels of HDL-c and reduced levels of TAG and VLDL-c (66).

*PPARs* are known as major transcriptional regulators of lipid and carbohydrate metabolism. Two studies based on the same RCT “RISCK Study” (29, 30) evaluated *PPAR*γ rs1801282 (Pro12Ala) and *PPAR*α rs1800206 (Leu162Val) in the same intervention, where the participants were randomly assigned to one of 3 diets to be consumed for 24 weeks: reference diet (high SFA), high MUFA diet and low-fat diet, all with equivalent PUFA intake. In the first study, 415 subjects were included, and only significant interaction between dietary PUFA:SFA ratio and genotype as a determinant of plasma concentrations of TC (*p* = 0.02), LDL-c (*p* = 0.002), and TAG (*p* = 0.02) was found; at a low PUFA:SFA ratio (0.33) mean TC and LDL-c concentrations in G (Ala12) carriers was significantly higher relative to non-carriers. There was also a significant trend in reducing plasma TAG (-50%) in G (Ala12) carriers as the PUFA:SFA ratio increased from 0.34 to 0.65 (*p* = 0.002). In the second study, also by AlSaleh et al. (29), both Pro12Ala *PPAR*γ and Leu162Val *PPAR*α had 45.7% lower levels of LDL-c after a high MUFA diet, compared to those with a low-fat diet (*p* = 0.005). Furthermore, *PPAR*γ G carriers in the reference diet group (high in SFA) had significantly higher TC and LDL-c than C (Pro12) carriers.

Even though this gene-diet interaction has not been described before, the study also concluded the results were related to an increased PUFA intake rather than the SFA intake. In the second study, several SNPs were genotyped in 466 subjects, and it was found that co-carriers of the *PPAR*γ rs1801282 G (Ala12) and *PPAR*α rs1800206 V (Val162) alleles had 45.7% lower LDL-c and 53.5% lower sdLDL (small dense LDL-c) as a proportion of LDL, after the high MUFA diet, compared to LFD.

The results discussed above complement what Rudkowska et al. (19) found in the Inuit cohort. Participants with C allele (wild type) of *PPAR*γ*2* rs10856710 had higher TC and LDL-c levels (*p* = 0.0175) when consuming a higher-TotFat and SatFat diet. These findings indicate that *PPAR*γ*2* rs10856710 C carriers benefit from a low SFA intake to lower TC and LDL-c levels.

##### Leptin Receptor

Leptin Receptor (*LEPR*) is responsible for coding the receptor for leptin, a hormone that regulates appetite and body weight. LEPR is also involved in other mechanisms like the regulation of fat metabolism. Mutations in this gene are associated with obesity (67).

Domínguez-Reyes et al. (25) analyzed the dietary fat intake of 100 subjects with normal weight and obesity using a frequency food consumption questionnaire. This study found that *LEPR* rs1137101 G carriers had two times higher risk of total cholesterol levels ≥200 mg/dl (OR = 2.1, 95 % CI 1.15–3.8; *p* = 0.008), AG/GG had a high risk of hypercholesterolemia (OR = 9.4, 95 % CI 2.1–41.5; *p* = 0.003). Furthermore, AG/GG of the rs1137101 polymorphism in the *LEPR* gene with an intake ≥12 g/day of saturated fatty acids have 3.8 times higher risk of cholesterol levels ≥200 mg/dl (*p* = 0.002) and 2.4 times higher risk of triglyceride levels ≥150 mg/dl (*p* = 0.02) than those with an intake <12 g/day SFA intake. Therefore *LEPR* rs1137101 AG/GG might benefit from a low SFA intake (<12 g/day SFA).

A possible explanation for this interaction is that when leptin signaling is defective in the liver, this may induce suboptimal protein expression levels of the lipolysis stimulator receptor, which may contribute to elevated postprandial lipemia (25, 68).

##### Hepatic Lipase (LIPC)

LIPC is a lipolytic enzyme that hydrolyzes TAG and phospholipids present in plasmatic lipoproteins like chylomicrons, LDL, and HDL lipoproteins, releasing free fatty acids and smaller lipoprotein particles (69).

Xu et al. (31) tested the effect of rs2070895 variant in *LIPC* on changes in blood lipids in response to various weight-loss diets: 2 diets were low fat (20%), and 2 diets were high fat (40%), or 2 diets were average protein (15%), and 2 diets were high protein (25%). After 2 years of intervention, they observed a long-term, cumulative pattern. Dietary fat significantly modified the genetic effects of rs2070895 on changes in serum TC, LDL cholesterol, and HD-c (*p* < 0.05). Data suggest that A carriers might benefit more in terms of lipid profile by eating a low-fat (20%), high-carbohydrate (55–65%) diet. A possible explanation for these findings comes from in animal studies showing that *LIPC* activity can be suppressed by a high-fat diet (70).

A similar interaction was found in the cohort study by Rudkowska et al. (19), where TT homozygotes of *LIPC* C-514T rs1800588 had higher TAG levels with a higher fat intake (*p* = 0.007).

Finally, Smith et al. (32) investigated dietary modulation of *LIPC* rs1800588 (-514 C/T) for lipids, comparing a high-fat Western diet and a low-fat traditional Hispanic diet consumption. The authors claim that the dietary patterns differed in total fat and fat composition (e.g., greater MUFAs contents in the Hispanic diet). In major allele carriers (CC/CT), HDL-c levels were higher following the Western diet than the Hispanic diet. Only major allele carriers benefited from the higher fat diet for HDL-c. However, in C carriers but not in T carriers, the Western diet was associated with higher LDL-c and higher TC in both phases of experimentation (high-fat Western diet and low-fat traditional Hispanic diet). *LIPC* rs1800588 CC/CT might benefit from an HFD (39% total fat) to raise HDL-c levels but may also raise LDL-c and TC levels. This result is somewhat controversial compared to Rudkowska et al. (19) and Xu et al. (31). It would be necessary to differentiate and prioritize between the benefits of an elevated HDL-c and an elevated LDL-c and their proportional contribution to total cholesterol.

##### Lipoprotein Lipase

Lipoprotein Lipase (LPL) is a crucial enzyme for lipid metabolism. It catalyzes the hydrolysis of triacylglycerols of chylomicrons and VLDL-c to provide free fatty acids for oxidation and, later, their use in the heart and other tissues and their storage in adipose tissue (71). In order to understand the relationship between rs13702T > C polymorphism in the 3'untranslated region of *LPL* and the fat intake on TAG, Corella et al. (33) studied 7,187 participants in the PREDIMED randomized trial that tested a MedDiet intervention (2 groups: one supplemented with extra-virgin olive oil and the other supplemented with nuts) compared with a low-fat diet control group. The rs13702T > C polymorphism was strongly associated with lower TAG in C carriers and interacted synergistically with dietary monounsaturated (*p* = 0.038) and unsaturated fat intake (*p* = 0.037), decreasing TAG at baseline. By 3 years, a gene-diet interaction was found in which the C allele was associated with a greater reduction in TAG after intervention with MedDiet, high in unsaturated fats (*p* = 0.025). The rs13702 C allele, which disrupts a microRNA-410 recognition element seed site resulting in a gain of function, was strongly associated with lower TAG levels, meaning that *LPL* rs13702 C carriers benefit from a high-unsaturated fat (30 g/day) MedDiet intervention in order to reduce TAG. According to Corella et al. (33), a possible explanation for this observation is that this interaction could result from the decrease in cellular oxidative stress associated with the consumption and content of unsaturated fats.

##### Fatty Acid-Binding Protein 2

The Fatty Acid-Binding Protein 2 (*FABP2*) gene encodes the intestinal fatty acid-binding protein (FABP), which is found in enterocytes and is involved in the intracellular transport of long-chain fatty acids. The Thr54 isoform (T allele) has been shown to have a two-fold greater binding affinity for the long-chain fatty acids. The Thr54 isoform has been associated with hypertriglyceridemia, increased body mass index (BMI), hyperinsulinemia, and insulin resistance.

De Luis et al. (35) showed that *FABP2* rs1799883 T carriers had a better metabolic response than obese patients with GG (Ala54Ala), with a decrease of total cholesterol, LDL-c, insulin levels, leptin levels, and HOMA-IR after weight loss with a high PUFA-hypocaloric diet (SAT = 21.8%, MUFAs: 55.5%, PUFAs = 22.7%, 7-g/day ω-6 fatty acids, 2-g/day ω-3fatty acids, and a ratio ω-3/ω-6 of 3.5). On the other hand, Martinez-Lopez et al. (34) showed that a moderate-fat diet (Fat:30%, SAT < 7%, MUFAs: 10–15%, and PUFAs: 10%, protein: 15%, CHO: 55%) had a positive effect on anthropometric and biochemical variables in overweight or obese subjects. This study showed that the T carriers responded better to a moderate-fat diet. In this way, Martinez-Lopez et al. (34) and De Luis et al. (35) suggested that high ingestion of PUFAs and MUFAs and a limiting dietary intake of SFA was beneficial for T carriers since the effects of the T allele and the diet contributed to the decrease of metabolic parameters as a result of the changes in the amount and type of fat intake. Under this condition, the isoform Thr54-I-FABP captures more “good” substrates (MUFAs and PUFAs) instead of saturated fats. Consequently, the Thr54 isoform (T allele) could be contributing indirectly to the expression of genes influenced by the peroxisome proliferator-activated receptor (72).

##### Fatty Acid Desaturase

Genes in the fatty acid desaturase (*FADS*) family of genes control the fatty acid metabolism pathway in the human body. This gene encodes a protein (desaturase enzymes) that controls and regulates the unsaturation of fatty acids by introducing double bonds between the defined carbons of the chain of the respective fatty acid. The protein encoded by the *FADS1* gene, for example, desaturates the PUFAs ω-3 and ω-6, acting as d-5 desaturase, so that EPA and arachidonic acid are formed (73).

Ching et al. (36) aimed to determine the interaction of ω-3 and ω-6 PUFAs with rs174547 in the *FADS1* gene in metabolic syndrome (MS). Chinese and Indian vegetarians were classified according to ω-3 and ω-6 intake. Results showed that middle LA (linoleic acid) and ALA (alfa-linoleic acid) groups with a middle intake of LA (5.87–8.19 g/day) had lower HDL-c levels (*p* < 0.05).

A possible explanation for this is that over-consumption of ω-6 may inhibit the production of ω-3 PUFAs, which reduces HDL-c level among those with TT homozygotes of rs174547 in the *FADS1* gene. Dietary intake of LA may interact with rs174547 in the *FADS1* gene to affect HDL-c level, which warrants the need to monitor the amount of LA and ALA in the regular diet of vegetarians. The health benefit of specific PUFAs may depend on individual *FADS1* genotypes or δ-5 desaturase activity, which is a rate-limiting enzyme in PUFAs biosynthesis encoded by the *FADS1* and *FADS2* genes (36, 74).

Lu et al. (37) examined whether genetic variations in the *FADS* gene cluster region interact with dietary intakes of ω-3 and ω-6 PUFAs to affect plasma total, HDL and non-HDL-cholesterol concentrations, and their relation with rs174546, rs482548, and rs174570 in the *FADS* gene cluster region, in 3575 participants in the second survey of the Doetinchem Cohort Study. Results showed significant associations between rs174546 genotypes and total and non-HDL-c concentrations in the group with a high intake of ω-3 but not in the low-intake group (*p* = 0.02). A significant gene-diet interaction was found as TC differed significantly according to rs175456 genotype (*p* = 0.02). The C carriers had higher TC concentrations, especially and only statistically significant in subjects with a high ω-3 PUFA intake (*p* = 0.006). Moreover, ω-6 PUFA intake also modified rs174546 genotype as the C allele was associated with higher HDL-c concentrations in subjects with a high intake (*p* = 0.004). No difference was seen for low ω-6 intake.

Although these studies do not provide the highest level of evidence due to their design, both results are congruent and show that polymorphisms in the *FADS1* and *FADS2* genes can affect non-HDL concentrations, leading to the conclusion that these polymorphisms benefit from a diet high in PUFAs, specifically, high in ω-6. The potential mechanism underlying this interaction between elevated desaturase activity and elevated CT or LDL-c levels is not yet entirely clear.

##### Fatty Acid Synthase

*FASN* codifies for a multifunctional enzyme that catalyzes palmitate synthesis from acetyl-CoA and malonyl-CoA into long-chain SFA (75).

Dolley et al. (38) hypothesized that positional candidate *FASN* polymorphisms and dietary fat might modulate LDL particle size. A Quebec Family Study (QFS) sample was studied, and five single nucleotide *FASN* polymorphisms were genotyped. Results showed that *FASN* rs4246444 was associated with LDL peak particle diameter (LDL-PPD) when fat intake was taken into account (*p* = 0.001). A carriers showed smaller LDL-PPD only when consuming a high amount of fat >35% total energy compared to the C carriers. The outcomes suggest that dietary fat intake may modify the effect of the *FASN* rs4246444 on LDL-PPD since *FASN* rs4246444-A carriers benefited from an LFD (<35% TE) in order to avoid LDL-PPD decrease and increase the number of LDL particles.

The diameter of small LDL-PPD is relevant because small LDL particles are considered more atherogenic as they can filter into vascular tissue and deposit in the subendothelial layer, contributing to the formation of atherogenic plaque and its consequences (76).

##### Tumor Necrosis Factor (TNF-α)

The superfamily of Tumor Necrosis Factor (*TNF*) genes codify multifunctional pro-inflammatory cytokines. One of its most studied variants, TNF-α, is a cytokine secreted by macrophages. This cytokine is implicated in multiple diseases, including those related to lipid metabolism, insulin resistance, arthritis, cancer, and others (77). Its increased expression in the adipose tissue of humans has been correlated with the degree of adiposity and associated with insulin resistance (39).

de Luis et al. (39) investigate the influence of G-308, rs1800629 of *TNF-*α on metabolic changes and weight loss secondary to high MUFA (Diet M) vs. high PUFA (Diet P) hypocaloric diets. In obese subjects, randomly allocated during 3 months to one of these diets for 3 months. Diet P resulted in reduced total cholesterol levels (*p* = 0.01), LDL cholesterol levels (*p* = 0.008) and TAG (*p* = 0.02). Therefore, G carriers (promoter variant of *TNF-*α gene) have a better metabolic response than obese carriers of A-allele consuming a high PUFA hypocaloric diet.

Gomez-Delgado et al. (23) obtained different results when examining whether the long-term consumption of a MedDiet, enriched in olive oil, compared with a low-fat diet, interacts with rs1800629 and rs1799964 SNPs at the *TNF-*α gene. In order to improve TAG, glycemic control, and inflammation markers. After 12 months of MedDiet, the decrease in TAG was statistically significant in GG subjects but not in A carriers (*p* = 0.005). Thus, the authors demonstrated that postprandial abnormalities associated with MS could be attenuated with high MUFA diets. One hypothesis to explain these findings could be related to the phenotypic flexibility associated with GG homozygotes MUFAs play a crucial role in maintaining phenotypic flexibility through TAG and inflammation homeostasis.

##### Max-Like Protein X Interacting Protein-Like

The *MLXIPL* gene encodes a transcription factor that, with the help of glucose, binds and activates elements for the response to carbohydrates in the promoters of TAG synthesis genes (78).

The effect of the MedDiet was analyzed by Ortega-Azorín et al. (40) in rs3812316 of the *MLXIPL* (Max-like protein X interacting protein-like) gene. A MedDiet intervention vs. a control diet for cardiovascular prevention was tested. The *MLXIPL* rs3812316 was associated with lower baseline TAG (*p* < 0.0001) and lower hypertriglyceridemia (odds ratio, 0.73 in G-carriers vs. CC homozygotes). This association was modulated by baseline adherence to MedDiet. When adherence to MedDiet was high, the protection was stronger and vice versa. Both the *MLXIPL* rs3812316 (*p* = 0.0001) and the MedDiet intervention (*p* = 0.030) were significantly associated with decreased TAG throughout the follow-up. Results suggest that MedDiet enhances the TAG-lowering effect of the *MLXIPL* rs381231.

The study was one of the first to report this gene-diet interaction. However, although the regulation of the *MLXIPL* remains unknown, several animal models have reported that the diet modulates the expression of this gene, thus affecting the metabolism of TAGs, which matches the outcome of the above-described study.

##### Transcription Factor 7 Like 2

The *TCF7L2* gene or transcription factor 7-like-2 encodes a protein that acts as a transcription factor with multiple functions and multiple polymorphisms. This gene is so broad that it is known as a pleiotropic gene (79).

Corella et al. (80) investigated the Transcription factor 7-like 2 (TCF7L2) rs7903146 polymorphism associations with type 2 diabetes, glycemia, blood lipids, and cardiovascular disease incidence. A randomized trial (two MedDiet interventions: group MedDiet + extra virgin olive oil (50 ml/d), group MedDiet + nuts (30 g/day), and a control group) with PREDIMED study sample was undertaken. Data were analyzed at baseline and after a median follow-up of 4.8 years. When adherence to the MedDiet was low, TT homozygotes had higher TC, LDL-c, and TAG (*p* = 0.005, *p* = 0.003, *p* = 0.046, respectively). Nevertheless, this increase was not observed (*p* = 0.605). Authors suggest that the overall MedDiet pattern rather than specific foods contribute to maintaining adequate TC, LDL-c, and TAG; therefore, TCF7L2 rs7903146 TT homozygotes recommendation is to follow a MedDiet pattern to maintain adequate levels of TC, LDL-c and TAG.

##### Fatty Acid Amide Hydrolase

Fatty Acid Amide Hydrolase (*FAAH*) gene encodes an integral membrane protein whose function is to hydrolyze bioactive amides to free fatty acids and ethanolamine (81).

Deluis et al. (41) investigated the role of *FAAH* rs324420 polymorphism on metabolic changes secondary to the administration of two kinds of diets. Basal and final measurements of 248 patients with obesity were compared after a 3-month period in which they received either an LFD or an LCD (low carbohydrate diet). With the LFD, TC and TAG decreased in the CC (wild-type homozygotes) and only decreased TC in AA homozygotes (*p* < 0.05). *FAAH* rs324420 CC benefits from consuming either an LFD to reduce TC and TAG or an LCD to decrease TC and LDL-c levels. The authors argue that a possible mechanism for these effects is that dysregulation of *FAAH* can lead to a higher hepatic and central endocannabinoids concentration, consequently leading to an increase in energy storage. With a hypocaloric diet, this regulation decreases and results in the observed improvement of the lipid profile.

##### Sterol Regulatory Element-Binding Transcription Factor 1

The *SREBP-1c* gene, as its name implies, encodes a transcription factor that binds to the sterol regulatory element (SRE1), which is one reason why it is found in the promoter of the LDL-c receptor gene and other genes involved in sterol biosynthesis. These genes have different transcription variants, including *SREBP-1a* and *SREBP-1c* (Genecards Org, 2021).

Zhang et al. (42) investigated the possible association between *SREBP-1c* rs2297508 polymorphism and lipid traits changes following a high-CHO/low-fat diet (15% fat and 70% CHO) for 6 days. Gender-specific changes in lipid profile emerged after treatment: levels of TC in males with GG homozygotes and C carriers decreased after HC/LF diet while female GG homozygotes also decreased levels of TC but increased TAG levels (*p* < 0.05). An HC/LFD might benefit *SREBP*-1c rs2297508 male and female GG homozygotes regarding the reduction of TC levels but may increase triglyceridemia in females.

#### Dietary Recommendations Related to Gene Variants and Carbohydrate or Dietary Fiber Intake

Carbohydrates should provide 40–60% of the total calorie intake in a balanced diet. Dietary fiber and sugars form carbohydrates, but their recommended intakes are specific and separate from carbohydrates. Dietary fiber consists mainly of structural polysaccharides that cannot be digested in the gastrointestinal tract of humans. There is ample evidence supporting that an adequate intake of fiber (20–35 g/day) helps to reduce blood cholesterol, prevent cardiovascular disease, and prevent constipation; there is also evidence about limiting the intake of free sugars to less than 10% of total energy intake, and even further reduction to 5%, as aiding measures for additional health benefits (58). Six studies were found in this systematic review that demonstrated a gene-diet interaction with carbohydrates or fiber (Table 2).

##### Cytochrome P450 Family 7 Subfamily A Member 1

The cytochrome P450 proteins are monooxygenases that catalyze many reactions in drug metabolism and the synthesis of cholesterol, steroids, and other lipids. This endoplasmic reticulum membrane protein catalyzes the first reaction in the cholesterol catabolic pathway of extrahepatic tissues, converting cholesterol to bile acids (82). Wang et al. (43) studied the effect of two different molecular weight β-glucan preparations on LDL-c and TC, in three groups of volunteers: 3 g/day high molecular weight (HMW), 3 g/day low molecular weight (LMW), and 5 g/day LMW, in mildly hypercholesterolemic adults, typified for *CYP7A1* rs3808607 G carriers. After a 5-week intervention, GG homozygotes seemed to benefit the most with 3 g HMW β-glucan/d administration and, more importantly, than TT homozygotes concerning TC and LDL-c levels (*p* < 0.05). This study provided knowledge regarding the influence of the molecular weight of β-glucans on their physiological effects, highlighting the importance of supplementing the diet with the polysaccharide and controlling its quality and molecular weight.

##### Apolipoprotein A5

Jang et al. (46) studied a dietary intervention program in *APOA5* rs662799 adult carriers with hypertriglyceridemia that consisted in replacing 1/3 of refined rice intake with legumes three times per day and an increased vegetable intake (6 units, 30–70 g/unit). A 12-week intervention significantly decreased TAG and increased HDL-c regardless of genotype (*p* = 0.002), although significantly lower TAG and higher HDL-c levels were recorded in *APOA5*−1121TT homozygotes compared with C carriers (*p* = 0.044). It is recommended that *APOA5* rs662799 TT homozygotes increase the intake of non-refined carbohydrates, 6 units of vegetables (30–60 g each) to reduce TAG and elevate HDL-c. *APOA5* was found to activate LPL *in vitro* and to accelerate TAG catabolism in animal studies. Therefore, reduced TAG levels in C carriers may be explained based on their reportedly reduced *APOA5* activity, which may result in decreased lipoprotein lipase (LPL) activity (46).

##### Cholesteryl Ester Transfer Protein

Gammon et al. (44) compared 82 hypercholesterolemic adults who were Cholesteryl ester transfer protein (*CETP)* Taq1B rs708272 carriers with two groups: a healthy diet and healthy diet plus two green kiwifruits per day. *CETP* Taq1B CC homozygotes (B1/B1) decreased TAG/HDL-c ratio after the kiwifruit intervention compared to the control group (*p* = 0.001). So, the final recommendation was to include two kiwi fruits per day to *CETP* rs708272 C carriers to improve TAG/HDL-c ratio. Elevated *CETP* activity results in enhanced TAG enrichment of HDL-c and LDL-c. *CETP* mass or activity was not measured in this intervention, but the difference in C carriers and T carriers (B2) could have been mediated by kiwifruit polyphenols decreasing *CETP* activity (83).

##### Alpha 2 Heremans-Schmid Glycoprotein

Similarly, Robinson et al. (45) performed another study involving 641 young Mexican healthy college students, where Alpha 2 Heremans-Schmid glycoprotein (*AHSG)* rs4917 carriers had higher levels of TAG with a higher carbohydrate intake (*p* = 0.004), specifically high in sugars (*p* = 0.05) and low in saturated fats (*p* = 0.01). *AHSG* is a gene that codes for Fetuin-A (FetA), a hepatokine and an adipokine that inhibits insulin receptor tyrosine kinase and thus reduces insulin sensitivity via decreased translocation of glucose transporter type 4; FetA has also been shown to bind saturated fatty acids and stimulate inflammatory cytokine release via toll-like receptor 4 (84). In this sense, Robinson et al. (45) detected that T carriers were associated with higher TAG levels in this cohort. However, the association was also strongly correlated with BMI. On the contrary, Song et al. (47) found in a group of Chinese healthy young college students that an intervention with a high carbohydrate (70% total calorie intake) LFD (15% total calorie intake) had favorable effects on TC, LDL-c, and HDL-c ratios, regardless of gender or *Apolipoprotein C3* (*APOC3*) SstI polymorphism (*p* = 0.001).

#### Dietary Recommendations Related to Gene Variants and Nutraceuticals and Dietary Supplements Intake

Nutraceuticals are defined as food or part of a food that provides health benefits, including preventing and treating disease beyond essential nutritional functions. Most nutraceuticals can be found in foods of plant origin; nevertheless, some of them can be found in animal foods (e.g., ω-3 fatty acids in fish). Besides, nutraceuticals come in different forms (whole foods, dietary supplements, or isolated compounds. In this way, dietary supplements are defined as “concentrated sources of nutrients (macronutrients, vitamins, minerals, enzymes, amino acids) or other substances with a physiological or nutritional effect to complement the diet” (85).

Both nutraceuticals and dietary supplements might positively impact consumer lipid traits when considering their genetic background.

##### Methylenetetrahydrofolate Reductase

The methylenetetrahydrofolate reductase (MTHFR) is an essential enzyme for folate metabolism, a necessary process for cell metabolism involving DNA, RNA, and protein methylation. It has also been shown that MTHFR C677T polymorphism is associated with hyperhomocysteinemia, hypertriglyceridemia, and elevated TC and LDL-c. In this context, Massa et al. (48) investigated the effect of a watermelon extract (*Citrullus lanatus*) supplementation (6 g/day) in adults carriers of *MTHFR* rsC677T or rs1801133 with diagnosed with dyslipidemia and found that total cholesterol and LDL-c levels were reduced after 6 weeks (*p* < 0.01). The most significant reduction was observed in CT and TT hetero and homozygotes. The effect may be related to the action of antioxidant compounds present in this fruit, such as lycopene, citrulline, and arginine (48).

##### Peroxisome Proliferator-Activated Receptor Gamma

Peroxisome proliferator-activated receptor gamma (PPARγ) agonists improve adipogenesis and insulin resistance, as well as dyslipidemia, without reducing food intake, and mutations in the *PPAR*γ are associated with obesity-related phenotypes. Lee et al. (49) analyzed the effects of *Kochujang* (Korean fermented soybean-based red pepper paste) consumed for 12 weeks (32 g/day: 39 g/day wet weight) by Korean subjects carriers of *PPAR*γ*2* C1431T rs3856806, with overweight or obesity. Significant plasma TAG reduction with *Kochujang* supplementation was not shown in the T allele rather than in the C carriers. Otherwise, the TAG/HDL ratio was lower with *Kochujang* supplementation in T carriers compared with the C carriers, since the plasma HDL-c and apoA-1 were also reduced in T carriers. These observations suggest a beneficial effect of Kochujang supplementation on the TAG and TAG/HDL weakened by the T mutant allele of the *PPAR*γ C1431T. Despite the concern of the high sodium content of *Kochujang*, it may have the potential to be developed as a functional supplement for the obese population (49).

##### Hepatic Lipase and Cholesteryl Ester Transfer Protein

Certain foods and beverages, like coffee, have been associated with beneficial health effects due to their phytochemical compounds, such as caffeine, chlorogenic acids, and trigonelline (86). Hsu et al. (50) analyzed 4,262 men and 4,813 women from the Taiwan Biobank, with polymorphisms in the genes of Hepatic lipase (*LIPC)* rs1800588 and Cholesteryl ester transfer protein (*CETP*) rs18007, both of which participate in lipoprotein metabolism. The cohort was divided into coffee drinkers (drank coffee > 3 times/week) vs. non-coffee drinkers. Only female coffee drinkers were significantly associated with higher HDL-c levels after adjusting for confounders, including rs1800588 (*LIPC*) and rs1800775 (*CETP*) variants. In this way, consumption of coffee and other caffeinated beverages may be beneficial for plasma levels of estrogen, sex hormone-binding globulin, and estradiol in women. However, information on the type of coffee consumed or drinking style was unavailable in the Taiwan Biobank database.

##### Cluster of Differentiation 36, Nitric Oxide Synthase 3, and Peroxisome Proliferator-Activated Receptor Gamma

Corella and Ordovás (87) suggested that *CD36, NOS3*, and *PPAR*γ genes have interactions with omega-3 (ω-3) fatty acids affecting the levels of blood lipids in intervention studies. Therefore, Zheng et al. (51) performed an RCT to study the gene-diet interaction of *CD36* (rs1527483)*, NOS3* (rs1799983), and *PPAR*γ (rs1801282) in response to supplements of omega-3 fatty acids (from fish oil: 2 g/day of C20:5ω-3 and C22:6ω-3, or flaxseed oil: 2.5 g/day of C18:3ω-3) in blood lipids of adults with T2D. After 180 days of treatment, omega-3 supplements from both sources interacted with *CD36* on TAG (*p* = 0.042) and with *PPAR*γ rs1801282 to reduce LDL-c levels (*p* = 0.02). These interactions were more pronounced with fish oil supplements, so the administration of 2 g/day of omega-3 (C20:5ω-3 and C22:6ω-3) is beneficial to reduce TAG or LDL-c in *CD36* (rs1527483) and *PPAR*γ rs1801282) carriers.

##### Polymorphism and Oil Supplements

Olano-Martin et al. (52) investigated the response of the plasma lipoprotein profile to eicosapentaenoic acid (EPA) vs. docosahexaenoic acid (DHA) in healthy adults with *APOE* rs429358, rs7412, E3/E3, and E3/E4. Four weeks of EPA-rich oil (ERO: 3.3 g EPA/day) and DHA-rich oil (DRA: 3.7 g DHA/day) supplementation resulted in significant TAG reductions (28 and 19%, respectively). The authors attribute this effect to an EPA/DHA mediated suppression of genes involved in the fatty acid synthesis and upregulation of those controlling beta-oxidation, resulting in an overall reduction in fatty acids available for TAG synthesis. In E4 carriers, DRO treatment resulted in a 10% increase in LDL-c (*p* = 0.029). For this reason, it is essential to monitor DRO supplementation in *APOE* rs429358 E3/E4. The findings generally agree with the greater VLDL to LDL conversion described in biokinetic studies in E4 carriers (70–80% vs. 50–60% in wild type E3/E3) (88). In this context, Carvalho-Wells et al. (24) determined the effect of dietary fat quantity and composition on both lipid and non-lipid cardiovascular disease biomarkers according to *APOE* genotype. The study involved 88 adults with an average BMI of 26 that were randomized into three isoenergetic diets: a low-fat diet, a high-fat/high SFA diet, and a high SFA supplemented with 3.45 g of DHA (HSF-DHA) per day. An overall diet effect was evident for all cholesterol fractions (*p* < 0.01), with no significant genotype x diet interaction. A genotype x diet interaction (*p* = 0.033) was evident for plasma TAG, with 17% and 30% decreases in *APOE* E3/E3 and *APOE* E3/E4 individuals, respectively, after the HSF-DHA diet compared with the low-fat diet. These observations indicate that E3/E3 and E3/E4 subjects benefit from 3.45 g DHA supplementation, even with a high SFA diet, in order to reduce TAG levels. *APOE* acts as a high-affinity ligand for the removal of VLDL remnants by the liver. The selective affinity of the apoE4 protein isoform for VLDL in contrast with the E2 and E3 isoforms, which have a preference for more lipid-poor large HDLs, may help explain the greater TAG lowering in *APOE* E4 carriers (89).

The efficacy of plant sterols (PS) as cholesterol-lowering agents is limited by a large heterogeneity across individuals, potentially because of genetic polymorphisms. MacKay et al. (53) evaluated the effect of ingesting 2 g of plant sterols/d or a placebo in mildly hypercholesterolemic adults preselected as exhibiting either high endogenous cholesterol synthesis or low endogenous cholesterol synthesis, as well as the presence of some polymorphisms: (Apolipoprotein E) *APOE* E3/E4, Cytochrome P450 Family 7 Subfamily A Member 1 *(CYP7A1)* rs3808607, *CETP* rs5882 and ATP-binding cassette sub-family G member 8 (*ABCG8)* rs4148217. Only *CYP7A1*-rs3808607 GT (*p* = 0.0006), GG (*p* = 0.0009), *APOE* E3 (*p* = 0.037) and especially E4 (*p* < 0.0001) were associated with LDL-c lowering in response to PS consumption, this specific finding could serve as potential predictive genetic markers to identify individuals who would derive maximum LDL-c lowering with PS consumption.

As it has been described throughout this review, both environmental and genetic factors impact lipid traits. Dumitrescu et al. (54) genotyped 23 lipid-associated variants in non-Hispanic white, non-Hispanic black, and Mexican-American subjects collected from the National Health and Nutrition Examination Surveys (NHANES). Gene-environment interactions between vitamin A or E and variants previously associated with HDL-c, LDL-c, and TAG levels were modeled. The most significant interaction was Apolipoprotein B (*APOB)* rs693 x vitamin E (*p* < 0.05) for LDL-c levels and proprotein convertase subtilisin/kexin type 9 *(PCSK9)* rs11206510 x vitamin A associated with LDL-c, both among Mexican-Americans. *PCSK9* is an enzyme that binds to low-density lipoprotein receptors (LDL receptors), which stop LDL-c from being removed from the blood, leading to an increase in blood levels of LDL-c. Thus, understanding the mechanism of the interaction between these lipid-associated variants and nutritional factors, such as serum vitamin E and A levels, is imperative to determining the etiology of a poor lipid profile and could, consequently, have implications in clinical care.

#### Dietary Recommendations Related to Gene Variants and Protein Intake

##### Fatty Acid-Binding Protein 2

Recent studies have suggested metabolic responses differences between the effects of various dietary approaches, including high protein diets.

As mentioned before, the Thr54 isoform (T allele) of *FABP2* rs1799883 has been associated with hypertriglyceridemia, and increased body mass index (BMI), hyperinsulinemia, and insulin resistance (90, 91).

de Luis et al. (55) investigated the influence of the variation Ala54Thr in the intestinal fatty acid-binding protein 2 gene (*FABP2*) on metabolic response secondary to the intake of a high-protein/low-carbohydrate (HP) vs. standard hypocaloric diet in subjects with obesity during 9 months. The two diets resulted in decreased anthropometric parameters in both genotype groups, but the improvement in most parameters was greater with HP than with the standard diet. With both diets and only in the wild genotype (GG homozygotes), TC and LDL-c levels decreased. Therefore, T carriers (Thr54) have a different metabolic response after weight loss than obese G carriers, with no effect on LDL-c or other metabolic parameters.

##### Uncoupling Protein 3

Uncoupling protein 3 *(UCP3*) belongs to a family of mitochondrial transporters that could uncouple oxidative phosphorylation by increasing the proton leak of the inner mitochondrial membrane. Decreased expression or function of UCP3 could reduce energy expenditure and increase energy storage as fat (92).

The effect of rs1800849 in the UCP3 promotor (−55C– > T) on cardiovascular risk factors and weight loss after a high protein/low carbohydrate vs. standard hypocaloric diet was studied as well by de Luis et al. (56). Results showed that with both diets and only in C carriers, total and LDL- c levels decreased. T carriers (mutant type) have a different response secondary to a standard hypocaloric diet than wild-type obese patients, and the authors hypothesized that the distribution of macronutrient and type of dietary fat could be involved in this different metabolic response. From this standpoint, they concluded that *UCP3* rs1800849 CC homozygotes benefit from a hypocaloric diet (either high protein/low carbohydrate or standard diet) to improve TC and LDL-c.

## Discussion

To the best of our knowledge, this is the first systematic review that collects information about the interaction of genetic variants with diet, seeking to identify nutrigenetic recommendations as potential tools to improve health through precision nutrition.

The objective of identifying and understanding gene-diet interactions is to enable guiding to feeding patterns based on individual and population genetic susceptibilities in contrast to the traditional “one size fits all” dietary recommendations. Although the concept of gene-diet interaction is attractive and its progress in recent years is encouraging, the lack of enough clinical interventions and replication is still a significant barrier limiting the acceleration of the field growth and its translation into practice.

The primary outcome of this review was the identification of potential nutrigenetic recommendations that demonstrate a strong interaction between gene-diet and circulating lipid variations. The difference between a recommendation and a requirement lies in the principle that a recommendation is population-based, and a requirement addresses the particularities of an individual, hence, making a dietetic treatment more appropriate and effective. The information identified in this review differentiates from generic or conventional recommendations for the dietary approach to dyslipidemias because the nutrigenetic recommendations are based on significant gene-diet interactions.

**Figure 2:**
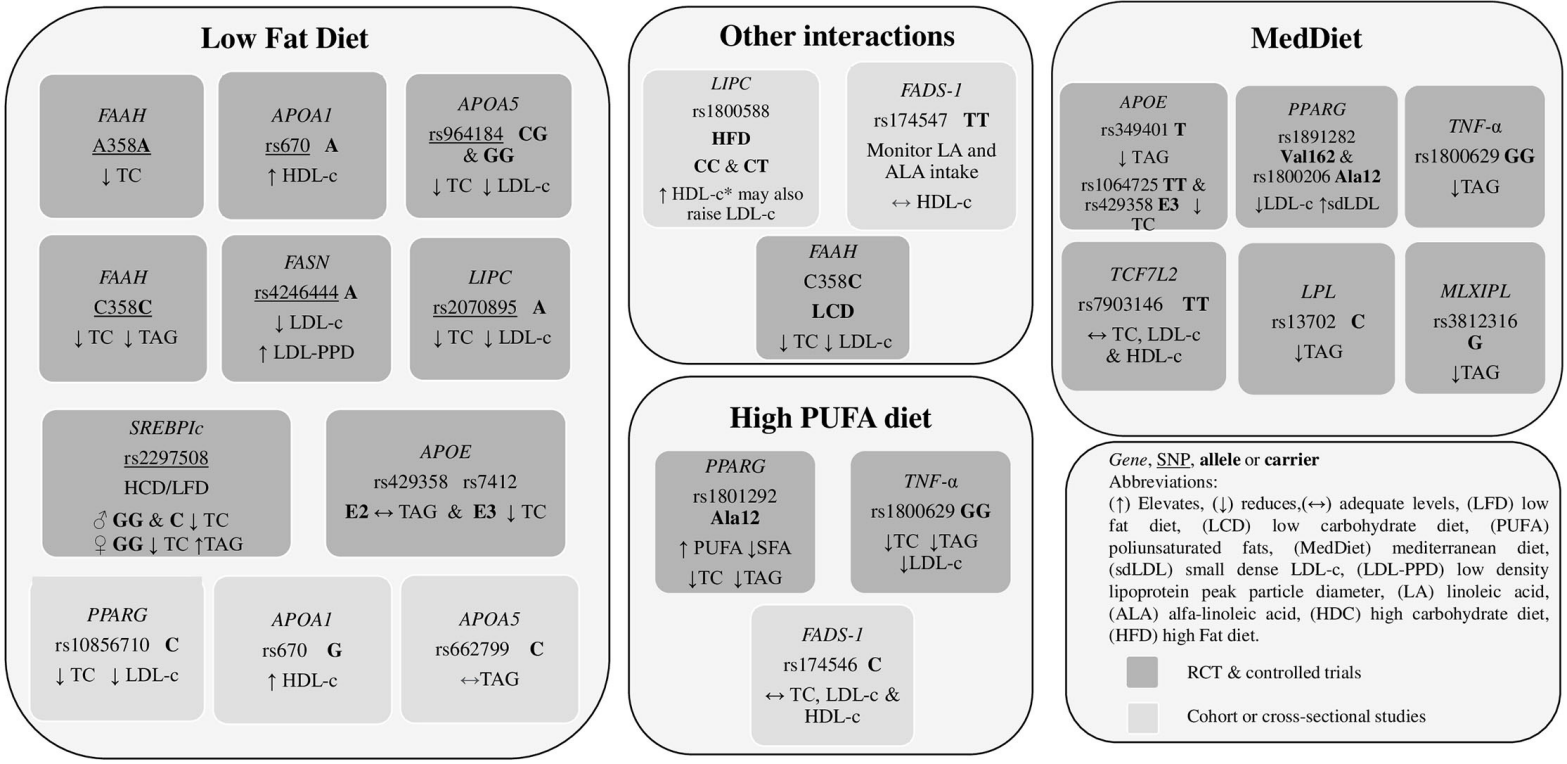
Dietary recommendations related to gene variants and dietary fat intake.

**Figure 3:**
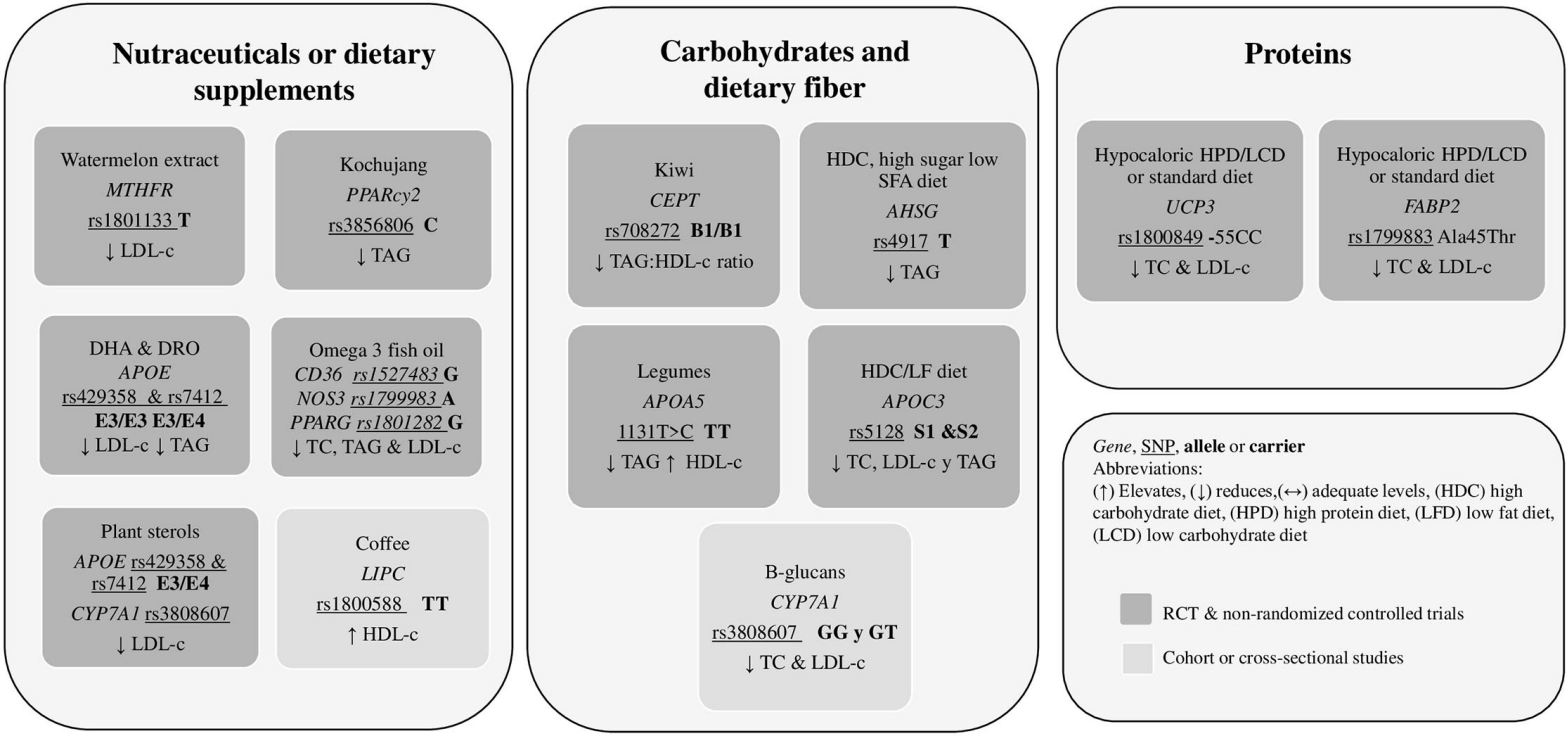
Dietary recommendations related to gene variants and diet components.

It was observed that the distribution of macronutrients intake is a crucial point in the interaction between body weight and metabolic response, considering the lipid profile or other biochemical markers. Dietary fat significantly modified the genetic effects of polymorphisms on changes in serum TC, LDL-c, and HDL-c. It was identified that the types and dosage of fatty acids included in the diet, whether they are PUFAS, MUFAS, or SFA, play a fundamental role in lipid metabolism. It should be noted that the dietary recommendations that benefit individuals with a particular genotype do not work in the same way for individuals with a different genotype. That is why genotyping individuals and populations is very relevant. Results of studies allowed us to observe how, through the intake of specific amounts and kinds of fats, carbohydrates/fiber, proteins, nutraceuticals or through the administration of precise dietary patterns made according to the anthropometric, physiological, and genetic needs of people, the decrease in biochemical parameters, mainly plasma lipids, was significant. Figure 2 summarizes the dietary recommendations related to gene variants and dietary fat intake, and Figure 3 summarizes the dietary recommendations related to gene variants and diet components identified throughout this review. The information collected in this review constitutes a finding that encourages and leads to creating nutritional strategies for preventing and controlling cardiometabolic diseases, a latent public health problem worldwide.

Concerning the consumption of carbohydrates, it is evident that the nutritional quality of these complex carbohydrate-rich and low glycemic index diets has a significant influence. The already known lipid-lowering effect of dietary fiber is reaffirmed. However, more research is needed to establish and understand the interaction of lipid genes with carbohydrates/fiber intake. It is also necessary to bear in mind that the general eating pattern helps maintain adequate levels of lipid traits and reduce cardiovascular risk.

Nevertheless, all recommendations can stand out from this review and highlight the importance of personalized nutrition. For example, gene-diet interactions with *FADS* genes were particularly appealing because there is a rise in current ω-3 y ω-6 vegetable sources like chia and flaxseed. However, gene-diet interactions found in Ching et al. (36) and Lu et al. (37) prove that these recommendations may not be adequate for people with polymorphisms in the *FADS* gene cluster family. Olano-Martin et al. (52) findings also highlight relevant gene-diet interactions with another famous and generic recommendation, fish oil supplementation with *APOE* and EPA-DHA administration, in which *APOE* rs429358 E3/E4 had an increase in LDL-c levels with DRO treatment which proves that this recommendation may not be adequate for everyone.

One of the strengths of this systematic review is that it addresses the evidence from studies that investigate the combined effect of genotype plus diet on health outcomes, which is essential for establishing evidence-based nutrigenetic advice. A limitation of this review was that due to the heterogeneity of the information obtained from the included articles, it was difficult to compare studies in terms of specific results and dietary recommendations suggested by the different authors. Another limitation was the non-inclusion of information since it came from articles that were evaluated with low methodological quality. However, the information transmitted can be considered enriching, recognizing that it is a pioneering review. Although the nutrigenetic area is still in its infancy, the available information is still considered scarce, heterogeneous and some findings are based on semi-quantitative data. It is crucial to stimulate debate on their usefulness and continue generating research as the knowledge on the subject increases.

The scientific community and community at large are facing the fact that nutrigenetics is unregulated, with few defined standards, there is a lack of educational resources or guidance for implementing the outcomes of nutrigenetic research. In this sense, it is considered that the proposed guidelines to evaluate scientific validity and evidence for genotype-based dietary advice by Grimaldi et al. (93) could be an effective tool to subsequently and individually evaluate each of the possible nutrigenetic recommendations included in this study as it is essential to consider aspects such as the study quality rating, type of gene x diet interaction (direct phenotype, intermediate phenotype, indirect phenotype), biological plausibility among others, to avoid misuse and to protect the public, personalized nutrigenetic advice and information should be based on clear evidence.

A proposal for further research is that the evidence obtained in this review could be a starting point for the future elaboration of “nutrition gene cards” that assess the evidence supporting specific gene × diet interactions and their relationship with a specific health outcome.

## Conclusion

The nutrigenetic recommendations identified may serve as dietary tools for the preventive treatment and control alterations in lipid metabolism. They are also potential tools for future research and a basis for creating nutrigenetic patterns and nutrigenetic portfolios containing specific dietary material for populations with different genetic and physiological characteristics. They can also be considered in potential strategies to treat public health problems related to dyslipidemias and several chronic degenerative diseases.

## Data Availability Statement

The original contributions presented in the study are included in the article/Supplementary Materials, further inquiries can be directed to the corresponding author/s.

## Author Contributions

YP-B: conceptualization, data curation, and writing—original draft preparation. IR-I and KG-B: conceptualization, visualization, methodology, data curation, and writing—review and editing. NP-N: writing—original draft. JT: writing—review and editing. SS-A: writing—review and editing and supervision. EM: conceptualization, visualization, writing—review and editing, supervision, funding acquisition, and project administration. All authors contributed to the article and approved the submitted version.

## Funding

The State Council of Science and Technology (COECYTJAL) funded this project with the Grant Number FODECIJAL-2019-8224.

## Conflict of Interest

The authors declare that the research was conducted in the absence of any commercial or financial relationships that could be construed as a potential conflict of interest.

## Publisher's Note

All claims expressed in this article are solely those of the authors and do not necessarily represent those of their affiliated organizations, or those of the publisher, the editors and the reviewers. Any product that may be evaluated in this article, or claim that may be made by its manufacturer, is not guaranteed or endorsed by the publisher.

## Abbreviations

ABCG8: ATP-binding cassette sub-family G member 8
AHSG: alpha_2_ Heremans-Schmid glycoprotein
APOA5: apolipoprotein A5
APOB: apolipoprotein B
APOC3: apolipoprotein C3
APOE: apolipoprotein E
CD36: cluster of differentiation 36,
CETP: cholesteryl ester transfer protein
CVD: cardiovascular diseases
CYP7A1: cytochrome P450 Family 7 Subfamily A Member 1
DHA: docosahexaenoic acid
EPA: eicosapentaenoic acid
FAAH: fatty acid amide hydrolase
FABP2: fatty acid binding protein 2
FADS: fatty acid desaturase
FetA: Fetuin-A
GI: glycemic index
GWAS: genome-wide association studies
HDL: high-density lipoprotein
HFD: high fat diet
IDL: intermediate density lipoprotein
LA: linoleic acid
LDF: low-fat diet
LDL: low density lipoprotein
LDL-PPD: LDL peak particle diameter
LIPC: hepatic lipase
LPL: lipoprotein lipase
MeSH: medical subject headings
MLXIPL: Max-like protein X interacting protein-like
MS: metabolic syndrome
MTHFR: methylenetetrahydrofolate reductase
MUFAs: monounsaturated fatty acids
NCBI: National Center for Biotechnology Information
NOS: Newcastle-Ottawa Scale
NOS_3_: nitric oxide synthase 3
PCSK9: proprotein convertase subtilisin/kexin type 9
PPARs: peroxisome proliferator activated receptors
PPARɤ2: peroxisome proliferator activated receptor gamma C1431T polymorphism
PRISMA-P: preferred reporting items for systematic reviews and meta-analyses protocol
PS: plant sterols
PUFAs: polyunsaturated fatty acids
RCT: randomized control trial
SFA: saturated fatty acids
SNP: single nucleotide polymorphism
SREBP-1c: sterol regulatory element binding transcription factor 1
TAG: triacylglycerols
T2D: type 2 diabetes
TC: total cholesterol
TCF7L2: transcription factor 7 like 2
TNF- a: tumor necrosis factor
UCP3: uncoupling protein 3
US NLM: United States National Library of Medicine.
