# Supplementary material for: Personalized Dietary Recommendations Based on Lipid-Related Genetic Variants: A Systematic Review
Source: Front Nutr. 2022 Mar 21;9:830283. doi: 10.3389/fnut.2022.830283 (PMC8979208; doi:10.3389/fnut.2022.830283)
Supplement: Supplementary file 1 [file Data_Sheet_1.docx]

***Supplementary Material***

**Contents:**

**Table S1.** MeSH terms used for search strategy

**Table S2.** Eligibility criteria of original studies included in this review according to (PICOS) format

**Table S3.** Assessment of the quality and level of evidence of 29 randomized control trials.

**Table S4.** Assessment of the quality and level of evidence of 4 cohort studies.

**Table S5.** Assessment of the quality and level of evidence of 6 cross-sectional studies.

| **Table S1. MeSH terms used for search strategy** | |
| --- | --- |
| “Dyslipidemia” | AND polymorphism AND diet  AND polymorphism AND carbohydrates  AND polymorphism AND protein  AND polymorphism AND fat  AND polymorphism AND PUFA  AND polymorphism AND MUFA  AND polymorphism AND SFA  AND polymorphism AND fiber  AND polymorphism AND vitamins  AND polymorphism AND minerals |
| “lipid polymorphism” | AND dietary  AND polymorphism AND diet  AND polymorphism AND carbohydrates  AND polymorphism AND protein  AND polymorphism AND fat  AND polymorphism AND PUFA  AND polymorphism AND MUFA  AND polymorphism AND SFA  AND polymorphism AND fiber  AND polymorphism AND vitamins  AND polymorphism AND minerals |
| “gene-diet” | AND dyslipidemia  AND interaction  AND lipid polymorphism |
| “gene dietary recommendation” OR “genetic dietary recommendation” | |

| **Table S2. Eligibility criteria of original studies included in this review according to (PICOS) format.** | | |
| --- | --- | --- |
|  | **Inclusion criteria** | **Exclusion criteria** |
| **Population** | Adults 18-85 years  Lipid abnormalities or dyslipidemia, insulin resistant, NAFLD, hypertension, diabetes, overweight or obese, metabolic related diseases, coronary heart disease, metabolic syndrome. | Children or adolescents >18 years without any other type of sickness not included in inclusion criteria (e.g. cancer, mental disorders, lupus, heart failure, hepatitis) or taking lipid-lowering drugs. |
| **Intervention or exposure** | Dietary intervention or diet analysis | Medical/ pharmacological or surgical intervention |
| **Comparator** | Lipid polymorphism | Polymorphisms not related to lipid-metabolism |
| **Outcomes** | Changes in lipids  (Total cholesterol, LDL-c cholesterol, HDL-cholesterol, VLDL-cholesterol and triglycerides) | Changes in other parameters: weight, BMI, waist circumference, glucose, insulin, HOMA-IR, leptin, adiponectin, etc. |
| **Study Design** | Randomized controlled trials, Non-randomized controlled trials, cohort or cross sectional | Meta analysis, systematic reviews, case-control studies, animal and laboratory studies (not involving humans) |

**Table S3. Assessment of the quality and level of evidence of 29 randomized control trials**.

| **Study** | **1**Is the study randomized? | **2-**Is the study double-blinded? | **3-** Losses and dropouts of subjects during follow-up are described? | **4-** Are the objectives of the study defined? | **5-** Are outcome measures clearly defined? | **6-** Is there a clear description of the inclusion and exclusion criteria? | **7-** Is the sample size justified (statistical power calculation)? | **8-** Is the description of the intervention clearly described? | **9-** Is there at least one control group or comparison group? | **10-** ​​Is the method used to assess adverse effects described? | **11-** Are the statistical methods used described? | **Total points** | **Quality evaluation** | **Level of evidence** |
| --- | --- | --- | --- | --- | --- | --- | --- | --- | --- | --- | --- | --- | --- | --- |
| Garcia-Rios et al. 2018 | 1 | 0 | 0 | 1 | 1 | 1 | 1 | 1 | 1 | 0 | 1 | 8 | Adequate | I |
| Shatwan et al. 2017 | 1 | 0 | 0 | 1 | 1 | 1 | 1 | 1 | 1 | 0 | 1 | 8 | Adequate | I |
| Griffin et al. 2018 | 1 | 0 | 1 | 1 | 1 | 1 | 1 | 1 | 1 | 0 | 1 | 9 | Adequate | I |
| Fallaize et al. 2016 | 2 | 0 | 1 | 1 | 1 | 1 | 1 | 1 | 1 | 0 | 1 | 10 | Adequate | I |
| Gomez-Delgado et al. 2014 | 2 | 0 | 0 | 1 | 1 | 1 | 1 | 1 | 1 | 0 | 1 | 9 | Adequate | I |
| Carvalho-Wells et al. 2012 | 1 | 0 | 0 | 1 | 1 | 1 | 1 | 1 | 1 | 0 | 1 | 8 | Adequate | I |
| Zhang et al. 2012 | 1 | 0 | 1 | 1 | 1 | 1 | 1 | 1 | 1 | 0 | 1 | 9 | Adequate | I |
| AlSaleh et al. 2011 | 1 | 0 | 0 | 1 | 1 | 1 | 1 | 1 | 1 | 0 | 1 | 8 | Adequate | I |
| Alsaleh et al. 2012 | 1 | 0 | 1 | 1 | 1 | 1 | 0 | 1 | 1 | 0 | 1 | 8 | Adequate | I |
| Xu et al. 2015 | 1 | 0 | 0 | 1 | 1 | 1 | 1 | 1 | 1 | 0 | 1 | 8 | Adequate | I |
| Corella et al. 2014 | 1 | 0 | 1 | 1 | 1 | 1 | 1 | 1 | 1 | 1 | 1 | 10 | Adequate | I |
| Martinez-Lopez et al. 2013 | 0 | 0 | 1 | 1 | 1 | 1 | 1 | 1 | 1 | 0 | 1 | 8 | Adequate | I |
| De Luis et al. 2012 | 1 | 0 | 1 | 1 | 1 | 1 | 1 | 1 | 1 | 0 | 1 | 9 | Adequate | I |
| De Luis et al. 2013 | 1 | 0 | 1 | 1 | 1 | 1 | 1 | 1 | 1 | 0 | 1 | 9 | Adequate | I |
| Ortega-Azorín et al. 2014 | 1 | 0 | 1 | 1 | 1 | 1 | 0 | 1 | 1 | 0 | 1 | 8 | Adequate | I |
| De luis et al. 2010 | 1 | 0 | 1 | 1 | 1 | 1 | 1 | 1 | 1 | 0 | 1 | 9 | Adequate | I |
| Zhang et al. 2011 | 1 | 0 | 1 | 1 | 1 | 1 | 0 | 1 | 1 | 0 | 1 | 8 | Adequate | I |
| Smith et al. 2017 | 1 | 2 | 1 | 1 | 1 | 1 | 0 | 1 | 1 | 0 | 1 | 10 | Adequate | I |
| Wang et al. 2016 | 1 | 0 | 1 | 1 | 1 | 1 | 0 | 1 | 1 | 1 | 1 | 9 | Adequate | I |
| Gammon et al. 2014 | 2 | 1 | 0 | 1 | 1 | 1 | 1 | 1 | 1 | 0 | 1 | 10 | Adequate | I |
| Olano-Martin et al. 2010 | 1 | 2 | 1 | 1 | 1 | 1 | 0 | 1 | 1 | 0 | 1 | 10 | Adequate | I |
| MacKay et al. 2015 | 1 | 0 | 1 | 1 | 1 | 1 | 1 | 1 | 1 | 0 | 1 | 9 | Adequate | I |
| Jang et al. 2010 | 0 | 0 | 1 | 1 | 1 | 1 | 0 | 1 | 1 | 1 | 1 | 8 | Adequate | I |
| Massa et al. 2016 | 1 | 2 | 1 | 1 | 1 | 1 | 0 | 1 | 1 | 0 | 1 | 10 | Adequate | I |
| Lee et al. 2017 | 1 | 2 | 0 | 1 | 1 | 1 | 0 | 1 | 1 | 0 | 1 | 9 | Adequate | I |
| Zheng et al. 2018 | 0 | 0 | 1 | 1 | 1 | 1 | 0 | 1 | 1 | 1 | 1 | 8 | Adequate | I |
| De Luis et al. 2015 | 1 | 0 | 1 | 1 | 1 | 1 | 1 | 1 | 1 | 0 | 1 | 9 | Adequate | I |
| De Luis et al. 2016 | 1 | 0 | 1 | 1 | 1 | 1 | 1 | 1 | 1 | 0 | 1 | 9 | Adequate | I |
| Song et al. 2011 | 1 | 0 | 1 | 1 | 1 | 1 | 0 | 1 | 1 | 0 | 1 | 8 | Adequate | II-1 |
| Quality evaluation according to Jadad scale. Level of evidence according to the USPSTF/ AHRQ. | | | | | | | | | | | | | | |

**Table S4.** Assessment of the quality and level of evidence of 2 cohort studies.

|  | **Selection** | | | | **Comparability** | | **Outcome** | | |  |  |  |
| --- | --- | --- | --- | --- | --- | --- | --- | --- | --- | --- | --- | --- |
| **Cohort study** | **Representativeness of the exposed cohort** | **Selection of the non exposed cohort** | **Ascertainment of exposure** | **Demonstration that outcome of interest was not present at start of study** | | **Comparability of cohorts on the basis of the design or analysis** | **Assessment of outcome** | **follow-up long enough for outcomes to occur** | **Adequacy of follow up of cohorts** | **Total** | **Quality evaluation** | **Level of evidence** |
| Lu et al. 2010 | ★ | ★ | ★ | ★ | | ★★ | ★ | ★ | ★ | 9 | Excellent | II-2 |
| Robinson et al. 2017 | ★ | - | ★ | ★ | | ★ | ★ | ★ | ★ | 7 | Good | II-2 |
| Quality evaluation according to Newcastle – Ottawa (NOS) quality assessment scale for cohort studies, Level of evidence according to the USPSTF/ AHRQ. | | | | | | | | | | | | |

**Table S5.** Assessment of the quality and level of evidence of 7 cross-sectional studies.

|  | **Selection** | | | | **Comparability** | **Outcome** | |  |  |  |
| --- | --- | --- | --- | --- | --- | --- | --- | --- | --- | --- |
| **Study** | **Representativeness of the sample** | **Sample size** | **Non- respondents** | **Ascertainment of the exposure** | **Subjects in different outcome groups are comparable** | **Assessment of outcome** | **Statistical test** | **Total** | **Quality evaluation** | **Level of evidence** |
| Domínguez-Reyes et al. 2015 | ★ | - | ★ | ★ | ★★ | ★★ | ★ | 8 | Good | II-3 |
| Sánchez-Moreno et al. 2011 | ★ | ★ | ★ | ★ | ★★ | ★★ | ★ | 9 | Excellent | II-3 |
| Ching et al. 2019 | ★ | ★ | ★ | ★ | ★ | ★★ | ★ | 8 | Good | II-3 |
| Dolley et al. 2011 | ★ | ★ | ★ | ★ | ★ | ★★ | ★ | 8 | Good | II-3 |
| Hsu et al. 2019 | ★ | ★ | ★ | ★ | ★★ | ★★ | ★ | 9 | Excellent | II-3 |
| Dumitrescu et al. 2012 | ★ | - | ★ | ★ | ★★ | ★★ | ★ | 8 | Good | II-3 |
| Rudkowska et al. 2013 | ★ | ★ | ★ | ★ | ★ | ★★ | ★ | 8 | Good | II-3 |
| Quality evaluation according to Newcastle – Ottawa (NOS) quality assessment scale adapted for cross-sectional studies. Level of evidence according to the USPSTF/ AHRQ. | | | | | | | | | | |
